# Investigation of a surface decorated Ag–MnO_2_@MGCN nanointerface towards membrane-free electrochemical Na^+^ ion sensing in sweat

**DOI:** 10.1039/d6ra04109d

**Published:** 2026-08-18

**Authors:** Nikita J. Patil, Ganesh Kumar Mani, Kamalakannan Kailasam, Murali Rangarajan, Parthasarathy Srinivasan

**Affiliations:** a Center of Excellence in NanoTechnology & Advanced Materials (CENTAM), Department of Electronics and Communication Engineering, Amrita School of Engineering, Amrita Vishwa Vidyapeetham Chennai Tamil Nadu 601103 India s_parthasarathy@ch.amrita.edu; b Center of Excellence in Advanced Materials and Green Technologies, Amrita School of Engineering, Amrita Vishwa Vidyapeetham Coimbatore India r_murali@cb.amrita.edu; c Department of Physics, Korea Advanced Institute of Science and Technology Korea; d Advanced Functional Nanomaterials, Institute of Nano Science and Technology (INST) Knowledge City, Sector-81, Manauli, SAS Nagar 140306 Mohali Punjab India kamal@inst.ac.in kkamal17@gmail.com; e Department of Chemical Engineering and Materials Science, Amrita School of Engineering, Amrita Vishwa Vidyapeetham Coimbatore India; f Gurukripa Electrolyzers Private Limited Coimbatore India

## Abstract

Noninvasive continuous electrolyte monitoring, including Na^+^ ion levels in sweat, is essential for real-time physiological assessment. The changes in sweat Na^+^ ion levels reflect underlying physiological conditions and might be used as a potential biomarker for electrolyte imbalances. In this work, we report an electrochemical Na^+^ ion switch based on a surface-anchored Ag–MnO_2_@mesoporous graphitic carbon nitride (MGCN) nanointerface. The results of morphological analysis revealed a hierarchical architecture consisting of MnO_2_ nanorods uniformly anchored to MGCN nanosheets and decorated with Ag nanoparticles that form a highly conductive and electroactive platform. Electrochemical analysis showed distinct oxidation and reduction peaks at 0.25 V and 0.08 V, respectively, indicating strong redox activity and efficient charge transfer upon interaction with Na^+^ ions. The Ag–MnO_2_@MGCN sensor showed a broad linear range from 0.1 to 150 mM, with a LOD of 0.06 mM, and a sensitivity of 9.62 µA mM^−1^ cm^−2^. The sensor also demonstrated excellent repeatability, stability and high recovery values ranging from 96.46% to 101.2% in artificial sweat samples, confirming its analytical reliability. The sensing mechanism is explained *via* the intercalation–deintercalation behaviour of Na^+^ ions within the MnO_2_ framework of Ag–MnO_2_@MGCN, while Ag nanoparticles and MGCN synergistically enhance charge transfer. These findings demonstrate the potential of the developed Ag–MnO_2_@MGCN nanointerface for the future development of membrane-free electrochemical sensors for Na^+^ detection in sweat.

## Abstract 2

Herein, selective sensing of sweat Na^+^ ions is achieved using an Ag–MnO_2_@MGCN nanocomposite through an intercalation–deintercalation mechanism.

## Graphical abstract

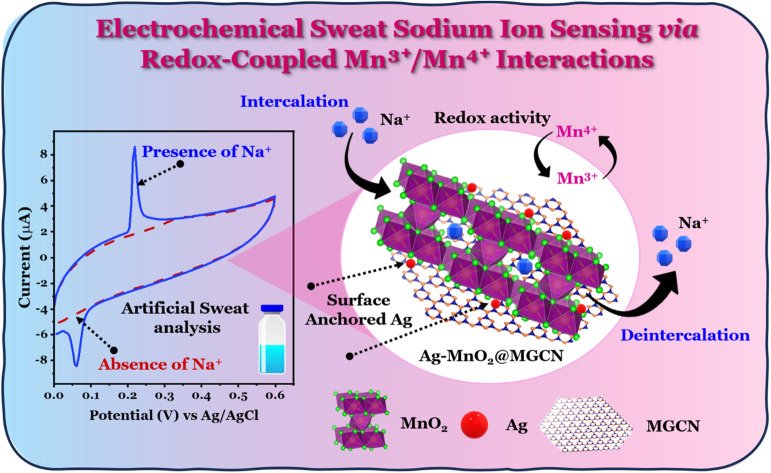

## 1 Introduction

The sodium ion (Na^+^) is a fundamental electrolyte in the human body, playing an essential role in maintaining fluid balance, nerve impulse conduction and muscle contraction. The normal plasma Na^+^ concentration lies within the range of 135 to 145 mmol L^−1^ and is primarily regulated by dietary intake, renal function, and hormonal control.^1^ Sweat Na^+^ levels serve as a direct, real-time indicator of electrolyte balance and hydration status, providing physiological insights.^2^ The Na^+^ concentration in sweat (10–100 mM) is closely related to blood Na^+^ levels, as changes in sodium intake directly impact its excretion through sweat.^3,4^ The plasma Na^+^ levels show delayed fluctuations in response to homeostatic regulation, and sweat Na^+^ levels show an immediate response to the fluctuation of electrolyte homeostasis.^5^ Increased Na^+^ concentration in sweat is typically associated with cystic fibrosis (CF), hypernatremia, and primary hyperaldosteronism, where increased sodium loss occurs due to impaired ion transport.^6^ In contrast, lower Na^+^ concentrations are associated with hyponatremia, adrenal insufficiency, chronic kidney disease, and overhydration, conditions that lead to sodium conservation as a compensatory mechanism.^7^ Therefore, probing Na^+^ in sweat enables a noninvasive platform for continuous monitoring with tailored sensing devices.^8^

Several analytical methods have been investigated to detect Na^+^ ions such as fluorescence spectroscopy, visible spectroscopy, colorimetric assays, and electrochemical sensors.^9–11^ Spectroscopic methods offer reliable and sensitive measurements, but the requirements of extensive sample preparation, complex instrumentation, and skilled operation makes them less suitable for practical applications. However, electrochemical sensors demonstrate high sensitivity, specificity, and surface functionalization capabilities, making them more suitable for rapid analysis and for continuous and non-invasive monitoring of Na^+^ in sweat.^12^

Yeung *et al.* reported the Na^+^ ion electrochemical sensor based on three-dimensional, graphene-based electrodes to enhance sensitivity and stability by forming a reducing water layer.^13^ In this design, the electroactive surface area was improved 7-fold over 2D electrodes with a sensitivity of 65.1 ± 0.25 mV per decade. Likewise, to achieve the multiplexed sensing of sodium (Na^+^), potassium (K^+^), and calcium (Ca^2+^) ions in sweat, Akshay *et al.* developed a flexible solid-state organic electrochemical transistor (ExG-SSOECT) composed of biocompatible semiconducting channels and ion-selective membranes (ISM), which offer high selectivity, fast response, and sensitivity.^14^

Despite ISM-based sensors offering high sensitivity, their inherent limitations, including labor-intensive synthesis, a lack of chemical robustness, and susceptibility to fouling in complex samples,^15^ make them less suitable for real-time analysis. Hence, there is a need to develop cost-effective, durable ISM-free Na^+^ sensors. In this context, Arash *et al.* developed a wearable potentiometric Na^+^ sensor utilizing Na_0.44_MnO_2_ as the sensing material, thereby eliminating the need for an ISM.^16^ The sensor exhibited a Nernstian response and a wide detection range (0.21–24.54 mmol L^−1^) for Na^+^, within the physiological range. Alex *et al.* developed an ISM-free Na^+^ sensor using iron(iii) phosphate-modified glassy carbon electrodes (GCE), employing stripping voltammetry for ion insertion and expulsion.^17^ This sensor exhibited good selectivity for Na^+^ ions and a linear response over 0.025–0.2 M, making it suitable for real-time detection in artificial sweat.

Although recent membrane-free Na^+^ sensing platforms have shown promising analytical performance, optimizing the electrochemical properties of the sensing materials remains a key challenge.^18–20^ Among the various electroactive materials, MnO_2_ has attracted significant attention because of its excellent redox activity, structural stability, and low-cost synthesis.^21^ However, its low intrinsic electrical conductivity limits charge-transfer kinetics and electrochemical response. Therefore, the rational integration of MnO_2_ with conductive nanomaterials is essential for enhancing electron transport and developing high-performance membrane-free Na^+^ sensing platforms.

With these developments, the present study aims to develop an ISM-free electrochemical Na^+^ sensor employing a surface-anchored Ag–MnO_2_@MGCN nanointerface for the first time. The observed sensing results indicate that a broader linear detection range with high sensitivity and specificity is achieved for sodium ions (Na^+^). The Na^+^ sensing scheme at the Ag–MnO_2_@MGCN nanointerface is attributed to the reversible intercalation and deintercalation of Na^+^ ions into the MnO_2_ lattice, which modulates redox activity and enhances voltammetric response.

## 2 Materials and methods

### 2.1 Materials

Uric acid (99%), *N*,*N*-dimethylformamide (DMF) (99.5%), and ascorbic acid (99%) were purchased from Sigma-Aldrich, Germany. Sulfuric acid (H_2_SO_4_) (98%), hydrochloric acid (HCl) (35%) and ammonium chloride (98%) were purchased from Emsure, Germany. Sodium hydroxide (NaOH) (98%) and potassium hydrogen phosphate (98%) were obtained from Loba Chemie, India. Potassium dihydrogen phosphate (99%) was purchased from S.D. Fine-Chem Limited, India. The other chemicals used for the synthesis were purchased from different brands as follows: silver nitrate (AgNO_3_) (99.8%) from Qualigens fine chemicals, glucose (extra pure) from SRL, sodium chloride (NaCl) (99%) from NICE chemicals, potassium chloride (KCl) (99%) and melamine from Himedia, lactate (92%) from Ranka, potassium permanganate (KMnO_4_) (99%), and urea (98%) from Avra, India. Deionized water was obtained from a Millipore system (18.2 MΩ cm^−1^).

### 2.2 Characterization

Surface morphological analysis of samples was studied using a Field Emission Scanning Electron Microscope (FE-SEM) with a ZEISS Gemini SEM 300 from Carl Zeiss, Germany. The structural analysis of the prepared samples was performed using an Ultima IV X-ray diffractometer (XRD) by Rigaku, Japan. UV-visible absorption spectroscopy was employed to investigate the optical properties using a JASCO V730 spectrophotometer (Japan). The Fourier Transform Infrared (FTIR) spectra were obtained using a Thermo Scientific Nicolet IS 10 IP spectrometer. The elemental composition and surface chemical states were analyzed using X-ray Photoelectron Spectroscopy (XPS) on a Thermo Fisher Scientific (Model: Nexa) apparatus equipped with a dual-anode X-ray source. Surface area analysis was measured using the BET (Brunauer–Emmett–Teller) method with a Quantachrome Autosorb automated gas sorption analyzer (India). Before analysis, the composite material underwent degassing for 5 h at 130 °C.

### 2.3 Synthesis of Ag–MnO_2_@MGCN nanocomposite

**Fig. 1:**
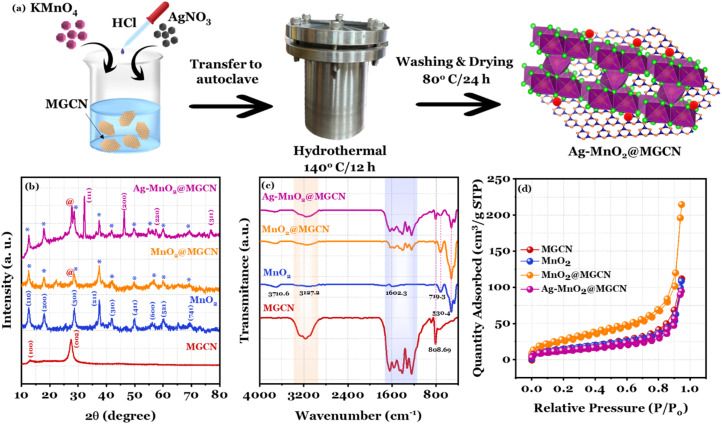
(a) Schematic illustration of the formation of Ag–MnO_2_@MGCN nanocomposite, (b) XRD patterns, (c) FTIR spectra and (d) N_2_ absorption–desorption isotherms of MGCN, MnO_2_, MnO_2_@MGCN, and Ag–MnO_2_@MGCN nanocomposite.

The synthesis of MGCN is comprehensively detailed in our previous work.^22^ Similarly, the Ag–MnO_2_@MGCN nanocomposite was synthesized using a modified hydrothermal method.^23,24^ Initially, 0.1 g of MGCN was dispersed in 40 mL of DI water and sonicated for 30 min to from uniform solution. Subsequently, 0.404 g of KMnO_4_ (2.56 mmol) was gradually introduced under continuous stirring, with the addition of 0.1 mmol of AgNO_3_ (17 mg) and 1.3 g of HCl. The reaction mixture was stirred again for 40 min, and then, 10 mL of DI water was added to ensure complete homogenization. Further resultant solution was transferred to a Teflon-lined autoclave and subjected to a hydrothermal reaction at 140 °C for 12 h. After cooling to RT, the product was washed several times with deionized water and ethanol to remove any unreacted precursors. Finally, the nanocomposite was dried in an oven at 80 °C overnight. For the synthesis of the MnO_2_@MGCN nanocomposite, the same procedure was followed, excluding the addition of AgNO_3_. The schematic representation of the synthesis procedure is shown in Fig. 1(a).

### 2.4 Electrochemical measurements

All electrochemical experiments in this study were conducted using a three-electrode system, consisting of a modified glassy carbon electrode (GCE) as the working electrode, an Ag/AgCl electrode as the reference and a platinum (Pt) wire as the counter electrode. The measurements were conducted using a Wave Driver 200 electrochemical workstation (Pine Research). The [Fe(CN)_6_]^3−/4−^ redox couple was employed as a standard electrochemical probe to assess the electron-transfer properties of the modified electrode surface. EIS measurements were conducted in a 5 mM [Fe(CN)_6_]^3−/4−^ solution containing 0.1 M KCl, using a 5 mV AC amplitude over a frequency range of 0.1 Hz to 100 kHz. Cyclic voltammetry (CV) was recorded in the same solution at a scan rate of 50 mV s^−1^ over a potential window of −0.2 to +0.6 V *vs.* Ag/AgCl. A 0.1 M phosphate buffer solution (PBS) was employed as the supporting electrolyte to ensure a stable environment for Na^+^ ion detection. For Na^+^ ion sensing, CV was performed over a potential range of 0.0 to 0.6 V *vs.* Ag/AgCl at a scan rate of 25 mV s^−1^. Differential pulse voltammetry (DPV) was carried out over the same potential range (0.0–0.6 V) using pulse parameters of 30 mV pulse height, 50 ms pulse width, 200 ms pulse period, and a 3 mV increment. Electrochemical studies were conducted in both PBS (pH 6.5) and human sweat as an electrolyte.

### 2.5 Preparation of Ag–MnO_2_@MGCN/MGCE sensor

The synthesized Ag–MnO_2_@MGCN nanocomposite was used to modify the GCE surface *via* drop-casting. GCE was first cleaned using α-alumina (α-Al_2_O_3_) polishing powder, followed by thorough rinsing with deionized water. To prepare the modification solution, 1 mg of Ag–MnO_2_@MGCN composite was dispersed in 1 mL of DMF solution, and the mixture was sonicated for 20 min to ensure optimal dispersion. A 1 µL aliquot of this composite suspension was then drop-casted onto the GCE surface, followed by exposure to infrared (IR) light for 5 min. The modified electrode was then dried under IR light for an additional 5 min before being used in further electrochemical studies.

## 3 Results and discussions

### 3.1 Structural analysis

The XRD patterns are presented in Fig. 1(b). The diffraction peaks were matched with JCPDS database [80-1917] and [44-0141] and they showed the polycrystalline nature of MGCN and MnO_2_. Two distinct peaks were observed for MGCN: one with low intensity at 2*θ* = 12.9°, corresponding to the (1 0 0) plane, attributed to the tri-*s*-triazine units; a further dominant peak at 2*θ* = 27.3° (0 0 2) shows interlayer stacking of the conjugated aromatic layers.^25^ For MnO_2_, characteristic diffraction peaks were observed at 2*θ* = 12.5°, 17.8°, 28.6°, 37.4°, 44.2°, 49.8°, 56.8°, 60.1°, and 68.5°, which correspond to the (1 1 0), (2 0 0), (3 0 1), (2 1 1), (3 1 0), (4 1 1), (6 0 0), (5 2 1), and (7 4 1) crystal planes, respectively.^26^ The highest intensity was observed for the (2 1 1) plane, indicating the preferential crystallographic orientation of MnO_2_. Moreover, the XRD pattern of MnO_2_@MGCN nanocomposite showed peaks of both MGCN (denoted by @) and MnO_2_ (marked by #). Notably, the MnO_2_ (3 0 1) and MGCN (0 0 2) peaks overlap at 27.3°, forming a broadened peak with a slight shift, which may be due to strong interfacial interaction between MnO_2_ and MGCN. The XRD pattern of Ag–MnO_2_@MGCN also showed peaks corresponding to both MGCN and MnO_2_, along with additional peaks for Ag at 2*θ* values around 31.7°, 45.3°, 57.1°, and 76.4°, which correspond to (1 1 1), (2 0 0), (2 2 0), and (3 1 1) planes of metallic silver, respectively.^27,28^ Strong diffraction from the Ag (1 1 1) plane and reduced intensities of the MnO_2_ peaks validate the successful formation of the ternary nanocomposite.

### 3.2 Functional group analysis

The FT-IR spectrum of MGCN showed an intense peak at 808 cm^−1^ attributed to the *s*-triazine ring (Fig. 1(c)).^29^ The stretching vibrations of N–(C)_3_ and C–NH–C bonds were found in the range of 1700–1200 cm^−1^, corresponding to specific peaks at 1638, 1558, 1459, 1439, 1400, and 1324 cm^−1^, attributed to the skeletal vibrations in the heptazine heterocyclic rings.^30,31^ In addition, three broad peaks at 3073, 3172, and 3257 cm^−1^ corresponded to the stretching vibrations of secondary amines (–N–H), primary amines (–N–H_2_), and O–H groups, respectively.^32,33^ In the case MnO_2_, the FT-IR spectrum showed key peaks below 750 cm^−1^, including 719, 530, and 463 cm^−1^ related to the metal–oxygen (Mn–O) bending vibrations of MnO_2_.^34,35^ A small peak observed at 1602 cm^−1^ due to O–H bending vibrations arises from adsorbed water on the surface of MnO_2_ nanorods during the KBr pelletization process.^36,37^ The FT-IR spectra of the MnO_2_@MGCN and Ag–MnO_2_@MGCN nanocomposites showed characteristic peaks of MGCN and MnO_2_, and a significant change in peak intensities and positions was observed.^38^ The composite contains MGCN, which was confirmed, ensuring the presence of a characteristic band at 808 cm^−1^. The dominant peaks of Mn–O bonds were obtained in the MnO_2_@MGCN composite, whereas the peak corresponding to the MGCN bonds was more dominant in the Ag–MnO_2_@MGCN composite, indicating a strong influence of Ag on the structure. The observed peak shifts and intensity variations further confirm the occurrence of electronic interactions between MGCN, MnO_2_, and Ag, confirming the successful synthesis of the Ag–MnO_2_@MGCN nanocomposite.^39^ These results are consistent with the expected structural modifications and interactions within the composite, validating the composite formation.

### 3.3 Surface area analysis and optical studies

N_2_ absorption–desorption isotherms of the prepared nanomaterials showed Type IV isotherms, characteristic of mesoporous structures (Fig. 1(d)).^40^ The surface area of MnO_2_@MGCN was found to be 91 m^2^ g^−1^, which is higher than that of bare MGCN (52 m^2^ g^−1^) and MnO_2_ (50 m^2^ g^−1^), indicating that the integration of MnO_2_ and MGCN significantly enhances the surface area. The pore diameter of MGCN was 3.8 nm, and the pore diameter of MnO_2_ was much larger (27.96 nm) (Fig. S1). The MnO_2_@MGCN composite exhibited a similar pore diameter of 28 nm, suggesting that MnO_2_ can be incorporated into the MGCN without losing the mesoporosity. The surface area of Ag–MnO_2_@MGCN was reduced to 41 m^2^ g^−1^, about half that of MnO_2_@MGCN, which indicates the successful incorporation of Ag nanoparticles in Ag–MnO_2_@MGCN composite. The reduction in surface area is a common behaviour for a metal nanoparticle-decorated surface, and can be explained by the partial blocking of the mesopores. Additionally, the agglomeration of Ag NPs, due to their high surface energy, further contributes to the decrease in surface area. This controlled modification of surface properties is a key requirement for electrochemical sensor applications, where a balance between surface area and conductivity is essential for performance.

The optical absorption profile of the prepared nanomaterials is presented in Fig. S2(a). The MGCN showed strong absorption within the 280 to 360 nm range, whereas the MnO_2_ and MnO_2_@MGCN exhibited a maximum absorbance within the 400–500 nm range.^41–43^ Interestingly, Ag–MnO_2_@MGCN nanocomposite showed the highest absorption peak at 322 nm.^44^ The optical band gaps of the synthesized nanomaterials were calculated from Tauc plots (Fig. S2(b–e)). The band gap values were 2.3, 2.47, 2.1, and 1.7 eV for MGCN, MnO_2_, MnO_2_@MGCN, and Ag–MnO_2_@MGCN, respectively. The decrease in band gap from the individual MGCN and MnO_2_ materials to their composites agrees with earlier reports on MGCN/metal-oxide systems, where interfacial coupling leads to band-gap narrowing. The further reduction in band gap for Ag–MnO_2_@MGCN is attributed to heterojunction formation within the ternary structure.^22,45^

### 3.4 Surface morphological analysis

**Fig. 2:**
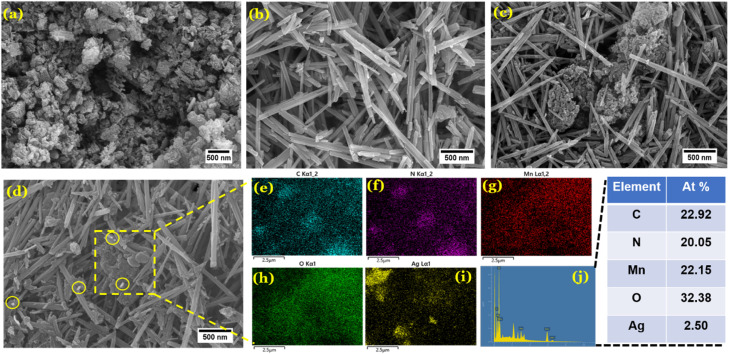
FESEM images of (a) MGCN, (b) MnO_2_, (c) MnO_2_@MGCN, and (d) Ag–MnO_2_@MGCN. Elemental mapping of Ag–MnO_2_@MGCN composite displaying the spatial distribution of (e) C, (f) N, (g) Mn, (h) O, and (i) Ag. (j) EDX spectrum confirms the elemental composition (scales are redrawn for better readability using Image-J software).

The pristine MGCN exhibits a structure composed of large, irregular, and crumbled mesoporous nanosheets, indicating a highly porous and non-uniform surface morphology (Fig. 2(a)). The morphology of MnO_2_ was primarily observed with uniformly distributed nanorods (Fig. 2(b)). MnO_2_@MGCN exhibit the presence of MnO_2_ nanorods along with the crumbled nanosheets morphology of MGCN (Fig. 2(c)). In the Ag–MnO_2_@MGCN composite (Fig. 2(d)), Ag nanoparticles were unevenly dispersed over the surface and showed with yellow circles. It suggests the morphological features of MnO_2_@MGCN were retained. Furthermore, the elemental analysis revealed the presence of C, N, Mn, O, and Ag in the sample (Fig. 2(j)). Additionally, the spatial distribution of these elements (Fig. 2(e–i)) demonstrates uniform dispersion of C, N, Mn, O, and Ag elements throughout the Ag–MnO_2_@MGCN composite.

**Fig. 3:**
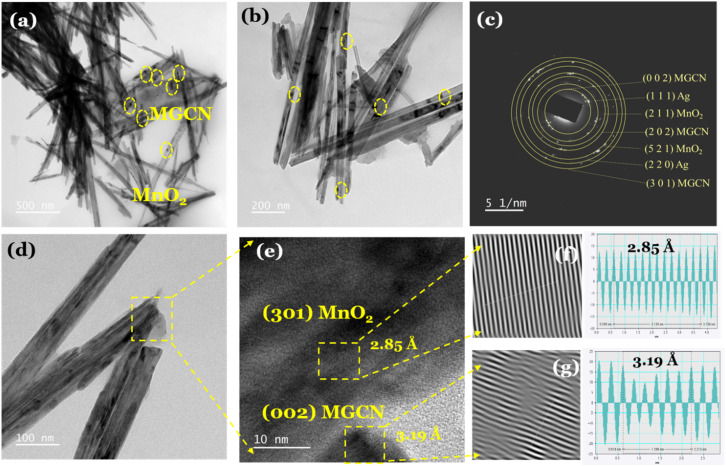
(a, b and d) TEM images of Ag–MnO_2_@MGCN, (c) SEAD pattern of Ag–MnO_2_@MGCN, (e) HRTEM image showing lattice fringes of MnO_2_ and MGCN, and (f–g) inverse FFT images with interplanar spacing estimation for MnO_2_ and MGCN.

TEM images of Ag–MnO_2_@MGCN (Fig. 3(a, b and d)), reveal that MnO_2_ nanorods were uniformly anchored and distributed with the crumbled MGCN nanosheets, forming a well-interconnected hybrid structure. Additionally, well-dispersed Ag nanoparticles were clearly observed in the MnO_2_@MGCN, confirming their successful incorporation within the hybrid matrix. The SEAD pattern (Fig. 3(c)) displays the diffraction rings corresponding to the (2 0 0), (2 0 2), and (3 0 1) planes of MGCN, indicating its layered graphitic nature. Distinct rings corresponding to the (2 1 1) and (5 2 1) reflections validate the polycrystalline characteristics of MnO_2_ nanorods. Additional rings indexed to the (1 1 1) and (2 2 0) planes indicate the crystalline nature of Ag nanoparticles. These diffraction features showed good alignment with the XRD results. HRTEM analysis (Fig. 3(e)) further revealed well-resolved lattice fringes corresponding to MnO_2_ and MGCN, with *d*-spacings of 2.85 Å and 3.19 Å, respectively, corresponding to the (3 0 1) plane of MnO_2_ and the (0 0 2) plane of MGCN.

### 3.5 Surface analysis

**Fig. 4:**
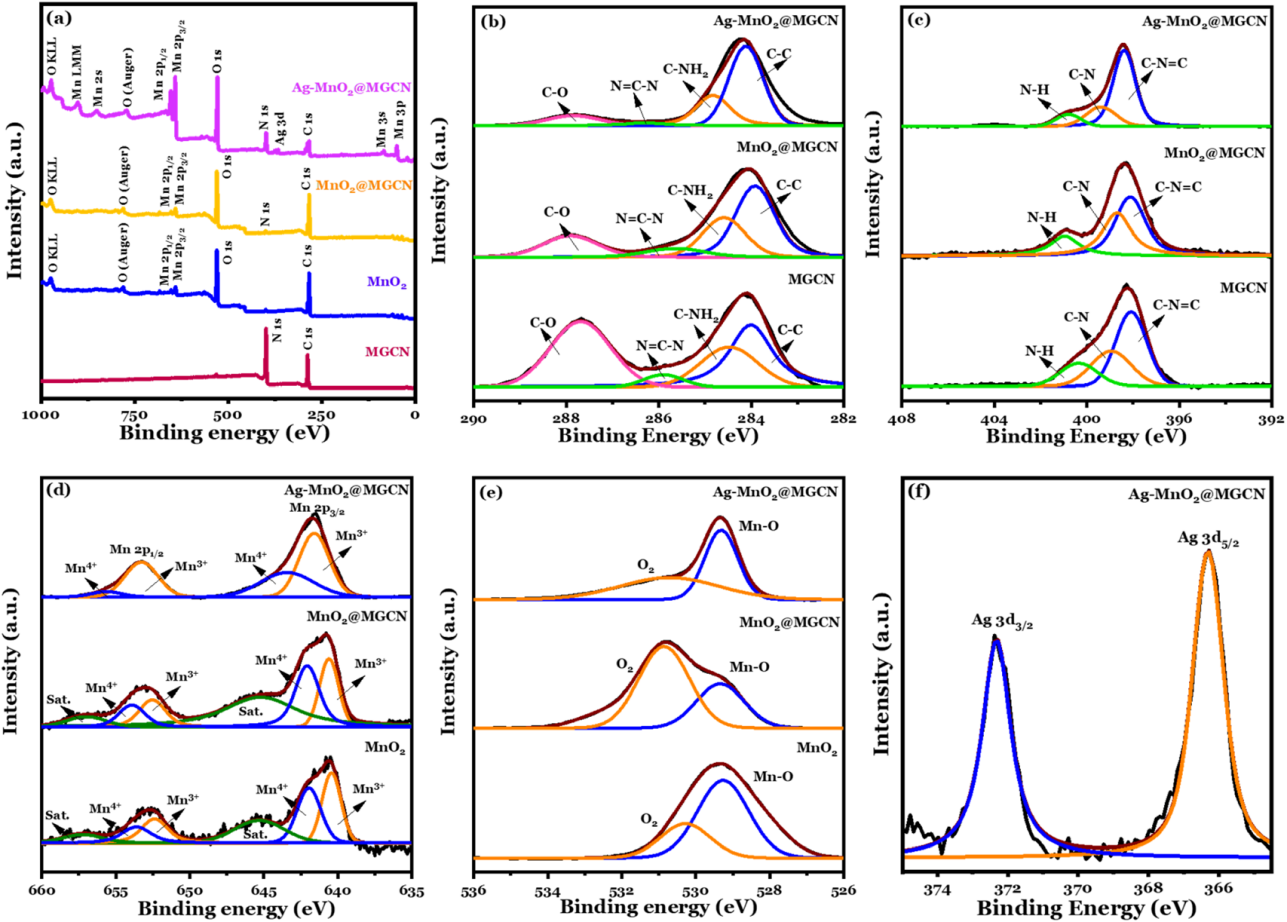
Comparative XPS analysis of MGCN, MnO_2_, MnO_2_@MGCN, and Ag–MnO_2_@MGCN: (a) survey spectrum, (b) C 1s spectra, (c) N 1s spectra, (d) Mn 2p spectra, (e) O 1s spectra, and (f) Ag 3d spectrum.

The surface chemical composition and electronic structure of the synthesized materials were investigated by XPS, and the corresponding spectra are presented in Fig. 4. The survey spectra (Fig. 4(a)) of MGCN, MnO_2_, MnO_2_@MGCN and Ag–MnO_2_@MGCN confirm the sequential formation of the heterostructure. Pristine MGCN exhibits characteristic C 1s and N 1s signals, whereas bare MnO_2_ displays only Mn 2p and O 1s peaks. Following the incorporation of MnO_2_ onto the MGCN framework, the simultaneous appearance of C, N, Mn, and O signals confirms the successful formation of the MnO_2_@MGCN composite.^46^ After Ag decoration, the emergence of Ag 3d peaks without any additional impurity signals verifies the successful incorporation of metallic Ag into the MnO_2_@MGCN heterostructure.^22,47^

The high-resolution C 1s spectra (Fig. 2(b)) of MGCN, MnO_2_@MGCN, and Ag–MnO_2_@MGCN can be deconvoluted into four peaks centred at 284.1 eV, 284.5 eV, 285.9 eV, and 287.6 eV, corresponding to C–C, C–NH_2_, N=C–H, and C–O species, respectively.^48,49^ Compared with pristine MGCN, the C 1s peaks exhibit slight binding-energy shifts upon MnO_2_ incorporation, with a further shift observed upon Ag decoration. These shifts indicate strong electronic interactions among Ag, MnO_2_, and the conjugated MGCN framework, suggesting the formation of an electronically coupled heterointerface *via* interfacial charge redistribution. Similarly, the high-resolution N 1s spectra (Fig. 4(c)) display three characteristic components located at 398.1 eV, 398.9 eV, and 400.4 eV, corresponding to C–N=C, C–N, and N–H bands, respectively.^50^ Compared with pristine MGCN, the gradual binding-energy shifts observed after MnO_2_ incorporation and subsequent Ag decoration further confirm the strong interaction between the nitrogen-rich MGCN framework and the deposited MnO_2_ and Ag nanoparticles. The electronic modulation of the nitrogen environment demonstrates that MGCN not only acts as a structural support but also participates in electronic coupling with the active components.

The Mn 2p spectra (Fig. 4(d)) of MnO_2_, MnO_2_@MGCN, and Ag–MnO_2_@MGCN exhibit the characteristic Mn 2p_3/2_ and Mn 2p_1/2_ doublets. Deconvolution of the spectra yields four peaks centred at 641.6 eV, 643.4 eV, 653.2 eV, and 655.5 eV, corresponding to Mn^3+^ (2p_3/2_), Mn^4+^ (2p_3/2_), Mn^3+^ (2p_1/2_), and Mn^4+^ (2p_1/2_), respectively, confirming the coexistence of mixed Mn oxidation states within the Mn oxide framework.^51^ The relative Mn^3+^ and Mn^4+^ contents were determined from the integrated areas of the fitted Mn 2p_3/2_ and Mn 2p_1/2_ components. Quantitative analysis revealed that the MnO_2_@MGCN nanocomposite contains 50.39% Mn^3+^ and 49.61% Mn^4+^, whereas the Ag–MnO_2_@MGCN nanointerface exhibits 67.84% Mn^3+^ and 32.16% Mn^4+^, indicating a pronounced increase in the Mn^3+^ following Ag incorporation. These changes in the Mn oxidation-state distribution suggest partial electron transfer from metallic Ag to the MnO_2_ lattice through interfacial charge redistribution, thereby shifting the Mn^3+^/Mn^4+^ equilibrium towards a higher Mn^3+^ species while preserving the mixed-valence character of the Mn-oxide.^52^

The O 1s spectra (Fig. 4(e)) exhibit peaks at 529.1 eV and 530.4 eV, corresponding to lattice oxygen (Mn–O) and surface-adsorbed oxygen/oxygen-deficient species, respectively.^53,54^ Compared with bare MnO_2_, slight binding energy shifts together with changes in the relative contribution of these oxygen species are observed after MGCN coupling and Ag decoration, indicating modification of the local oxygen coordination environment through interfacial electronic interaction. The coexistence of lattice oxygen and oxygen-deficient species is beneficial for enhancing the electronic properties of the composite by facilitating charge transport across the heterointerface. The Ag 3d spectrum (Fig. 4(f)) revealed the presence of two peaks at 366.2 eV (Ag 3d_3/2_) and 372.3 eV (Ag 3d_5/2_) and a spin energy separation of 6.1 eV, confirming the presence of Ag in its metallic state within the composite.^47^

### 3.6 Electrochemical sensing studies

#### 3.6.1 Impedance analysis of modified electrodes

**Fig. 5:**
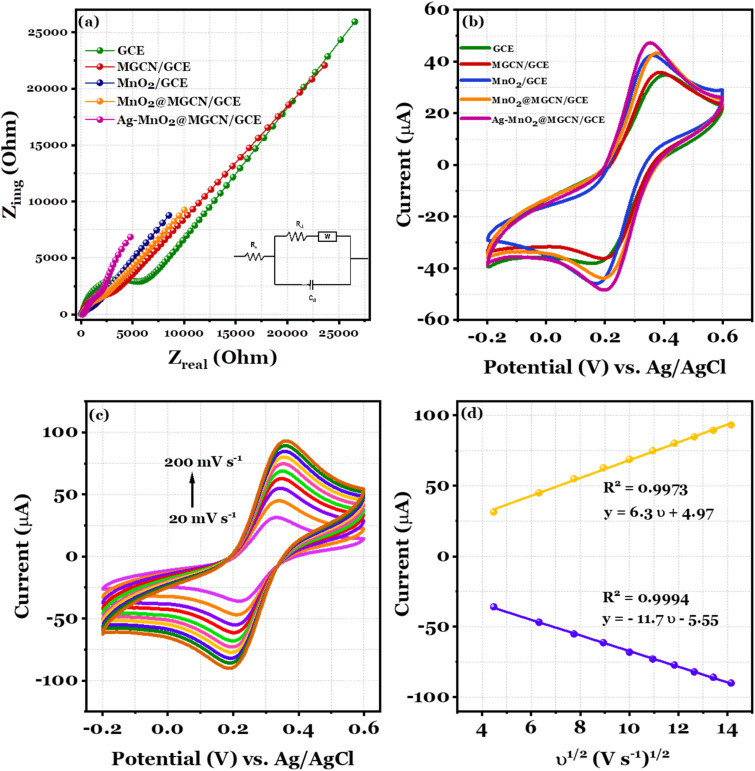
(a) EIS response of bare GCE, MGCN/GCE, MnO_2_/GCE, MnO_2_@MGCN/GCE, and Ag–MnO_2_@MGCN/GCE in 5 mM Fe(CN_6_)^3−/4−^ in 0.1 M KCl: inset of Randel's circuit (b) CV curves of different GCE modified electrode in 5 mM Fe(CN_6_)^3−/4−^ in 0.1 M KCl (c) different scan rate analysis (20–200 mV s^−1^) at Ag–MnO_2_@MGCN/GCE electrode, (d) linear plot of scan rate (mV s^−1^) and redox current (µA).

The interfacial charge-transfer properties of all electrodes, including MGCN/GCE, MnO_2_/GCE, MnO_2_@MGCN/GCE, and Ag–MnO_2_@MGCN/GCE, along with bare GCE, were analyzed using EIS (Fig. 5(a)). The impedance spectra were fitted using the Randles equivalent circuit (inset of Fig. 5(a)), and the extracted charge-transfer resistance (*R*_ct_) values are shown in Table S1. The bare GCE had the highest *R*_ct_ value, suggesting slow interfacial electron transfer kinetics. Further, a significantly decreased *R*_ct_ value was observed for the MGCN/GCE electrode, indicating that the graphitic carbon nitride framework effectively facilitates charge transport by forming a conductive and electrochemically active interface. The incorporation of MnO_2_ further lowers the *R*_ct_ value due to the presence of electroactive Mn centres that facilitate faradaic charge-transfer processes. Although the *R*_ct_ value of MnO_2_@MGCN/GCE remained comparable with MnO_2_/GCE, hybrid structures offer improved dispersion of MnO_2_ on the MGCN nanosheets, improving the electroactive sites and efficient electrical contact between the two components.

The Ag–MnO_2_@MGCN/GCE showed the lowest *R*_ct_ values among all the investigated electrodes, indicating the most favorable charge-transfer characteristics. The decreased *R*_ct_ is due to the high electrical conductivity of Ag nanoparticles, which form efficient electron transport pathways between MnO_2_ and the MGCN framework. The synergistic interaction of Ag, MnO_2_, and the MGCN framework significantly enhances the charge-transfer kinetics, which is directly correlated with the superior electrochemical sensing performance of the Ag–MnO_2_@MGCN/GCE electrode toward Na^+^ detection.

#### 3.6.2 Electrochemical behaviour of modified electrodes

CV analysis of bare GCE, MGCN/GCE, MnO_2_/GCE, MnO_2_@MGCN/GCE, and Ag–MnO_2_@MGCN/GCE are presented in Fig. 5(b). Redox peaks were observed for all modified electrodes, with Ag–MnO_2_@MGCN/GCE exhibiting the highest peak, indicating the catalytic effect. The enhanced catalytic activity of Ag–MnO_2_@MGCN/GCE is attributed to the following factors: (i) MnO_2_ nanorods provide multiple oxidation states (Mn^3+^ (2p_3/2_), Mn^4+^ (2p_3/2_), Mn^3+^ (2p_1/2_), and Mn^4+^ (2p_1/2_)), offering increased active sites, (ii) the incorporation of Ag NPs on the nanocomposite facilitates increased electron transfer rate, and (iii) heterojunction formation between MGCN and Ag–MnO_2_ improves both catalytic activity and electrical conductivity. Fig. 5(c) shows the CV response of Ag–MnO_2_@MGCN/GCE at various scan rates (20–200 mV s^−1^), which exhibits a strong redox current response and low peak potential difference, indicative of rapid electron transfer. Fig. 5(d) presents the linear relationship between the square root of the scan rate and the redox peak currents. The electrochemically active surface area (ECSA) of the redox reaction was calculated using the Randles–Sevcik equation:^55,56^

1 *I*_pa_ = (2.69 × 10^5^)*n*^3/2^*D*^1/2^*Aν*^1/2^*C*

where, *I*_pa_ (peak current), *n* (number of electrons involved in the reaction), *D* (diffusion coefficient of [Fe(CN)_6_]^3−/4−^), *A* (active surface area), *ν* (scan rate), and *C* (concentration of the [Fe(CN)_6_]^3−/4−^ solution), respectively. The highest ECSA was observed for Ag–MnO_2_@MGCN/GCE (0.065 cm^2^), followed by MnO_2_@MGCN/GCE (0.059 cm^2^), MnO_2_/GCE (0.057 cm^2^), MGCN/GCE (0.049 cm^2^), and bare GCE (0.046 cm^2^). Notably, Ag–MnO_2_@MGCN/GCE showed nearly a two-fold increased ECSA compared to bare GCE, demonstrating that the modification significantly enhances the electrode surface by enabling more active sites, a larger surface area, and faster electron transfer kinetics. These improvements make Ag–MnO_2_@MGCN/GCE a highly promising platform for Na^+^ ions detection.

#### 3.6.3 Electrochemical detection of Na^+^ ions at different modified electrodes

**Fig. 6:**
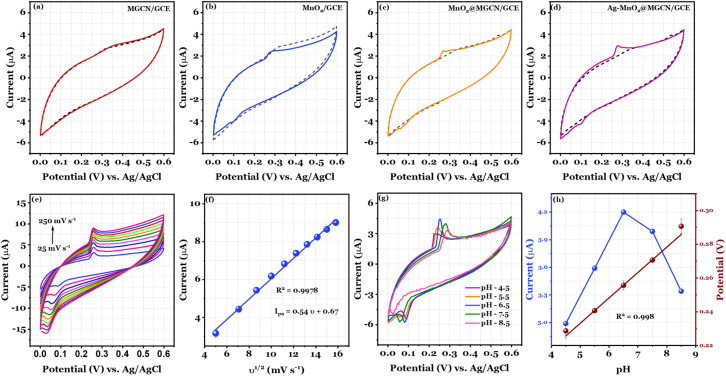
CV curves of (a) MGCN/GCE, (b) MnO_2_/GCE, (c) MnO_2_@MGCN/GCE, and (d) Ag–MnO_2_@MGCN/GCE in 0.1 M PBS (pH-6.5), in the absence (dotted line) and presence (solid line) of 5 mM Na^+^ ions at the scan rate of 25 mV s^−1^. (e) CV curves at a scan rate of 25–250 mV s^−1^ in 10 mM Na^+^, (f) calibration plot of *ν*^1/2^*vs. I*_pa._ (g) CV curves recorded in different pH levels from 4.5 to 8.5, and (h) the trend of pH *vs.* peak current and peak potential of Ag–MnO_2_@MGCN/GCE.

The electrochemical properties of the different modified electrodes: bare GCE, MGCN/GCE, MnO_2_/GCE, MnO_2_@MGCN/GCE, and Ag–MnO_2_@MGCN/GCE, were studied in 0.1 M PBS (pH – 6.5) with 5 mM Na^+^ ions. The CV curves were recorded in the presence and absence of Na^+^ ions to evaluate the Na^+^ sensing performance of each electrode. The CV profile of bare GCE and MGCN/GCE electrodes remained unchanged even in the presence of Na^+^ ions (Fig. S3 and 6(a)). This indicates electrodes has a less ability to facilitate Na^+^ ion intercalation. On the other hand, the MnO_2_/GEC, MnO_2_@MGCN/GCE and Ag–MnO_2_@MGCN/GCE electrodes exhibit clear redox peaks Fig. 6(b–d), confirming their ability to promote the electrochemical detection of Na^+^ ions. The Ag–MnO_2_@MGCN/GCE electrode shows higher redox current than MnO_2_/GCE and MnO_2_@MGCN/GCE electrodes, which indicates excellent sensing performance.

**Scheme 1:**
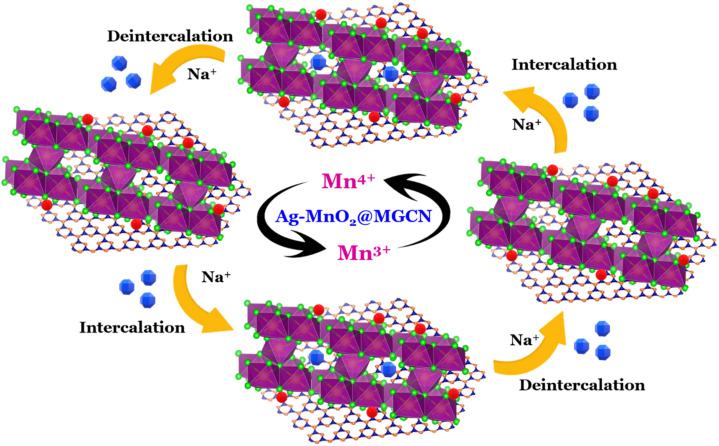
Intercalation–deintercalation-based sensing mechanism of Na^+^ ions at the Ag–MnO_2_@MGCN nanointerface.

The improved sensing performance of Ag–MnO_2_@MGCN/GCE arises due to the dual-contribution mechanism. (i) Mn^3+^/Mn^4+^ redox-coupled Na^+^ intercalation–deintercalation: the MnO_2_ lattice plays a central role by enabling reversible insertion and extraction of Na^+^ ions with a transition in the oxidation state of Mn.^57^ In the anodic (oxidation) sweep, Na^+^ ions are expelled from the MnO_2_ host lattice by oxidizing Mn^3+^ → Mn^4+^ (Scheme 1):2
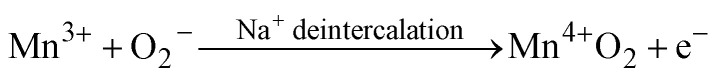


Conversely, during the cathodic (reduction) sweep, Na^+^ ions re-enter the MnO_2_ lattice, and Mn^4+^ is reduced back to Mn^3+^:3
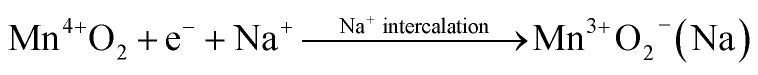


This reversible transition is represented as:

4 MnO_2_ + Na^+^ + e^−^ ↔ MnOO(Na)

The porous nanorod structure of MnO_2_ offers a large number of interstitial sites, short diffusion paths and facilitates fast Na^+^ ion transport and the reversible Mn^3+^/Mn^4+^ redox reaction. This redox behaviour is controlled by intercalation–deintercalation and pseudocapacitive charge-storage mechanisms, which were explained in MnO_2_-based Na-ion charge storage systems.^58,59^ As discussed in the XPS analysis, Ag incorporation induces interfacial charge redistribution, resulting in an increased Mn^3+^ oxidation state within the MnO_2_ framework. The increased Mn^3+^ content enhances the density of electrochemically active Mn^3+^/Mn^4+^ redox centres, thereby facilitating reversible Na^+^ intercalation–deintercalation by providing additional charge-compensation sites during Na^+^ insertion and extraction. This electronic modulation promotes faster redox kinetics and contributes to the enhanced faradaic current response observed for Ag–MnO_2_@MGCN/GCE.

In this work, intrinsic redox-mediated Na^+^ ion insertion is used in a sensing-oriented regime, where the reversible interaction of Na^+^ ions with the MnO_2_ lattice modulates the faradaic current response. The current variation observed is directly related to the concentration of Na^+^, and thus provides a sensitive and quantitative electrochemical detection without bulk energy storage effects.^58,59^ To further support the proposed sensing mechanism, EIS measurements were performed before and after Na^+^ exposure (Fig. S4). The fitted Nyquist plots exhibited only a slight variation in the electrochemical parameters, with the solution resistance (*R*_s_) increasing from 17.44 Ω to 26.33 Ω and *R*_ct_ changing from 7087.2 Ω to 7095.8 Ω after Na^+^ exposure. The negligible variation in *R*_ct_ indicates that Na^+^ interaction with the MnO_2_ framework preserves interfacial electron-transfer kinetics while supporting the reversible Na^+^ insertion–deintercalation process coupled with the Mn^3+^/Mn^4+^ redox transition.^60–62^ Furthermore, to provide complementary structural evidence, an additional Na^+^ treatment experiment was performed using the Ag–MnO_2_@MGCN nanocomposite. Briefly, 100 mg of the Ag–MnO_2_@MGCN powder was dispersed in 50 mL of DI water, followed by the addition of 0.1 M NaCl. The suspension was continuously stirred for 24 h at 60 °C to promote Na^+^ interaction with the MnO_2_. After treatment, the sample was thoroughly washed, dried and characterized by XRD and FTIR (Fig. S5). Compared with the pristine material, the Na^+^-treated sample exhibited no additional diffraction peaks, while only slight peak shifts and minor intensity variation were observed, indicating that the MnO_2_ crystal framework remained the same after Na^+^ interaction.^63^ Similarly, the FTIR spectra retained the characteristic vibrational band in the 2800–3400 cm^−1^ regions, suggesting a minor modification of the local chemical environment while preserving the host framework.^64^ These complementary electrochemical and structural results are consistent with previously reported MnO_2_-based Na^+^ intercalation–deintercalation-assisted Mn^3+^/Mn^4+^ redox mechanism.^65^

(ii) Surface charge transfer enhancement using Ag and MGCN: the electron transfer kinetics are enhanced using Ag nanoparticles and MGCN. Ag NPs serve as highly conductive catalytic sites that enable fast electron transfer between Mn redox centres and the electrode. Meanwhile, MGCN offers a high surface area, π-conjugated network with the capability of attracting Na^+^ ions through electrostatic interactions, which improves electronic conductivity.^22,66^ These effects collectively reduce the overall *R*_ct_, as demonstrated in Fig. 5(a), which is lower than MnO_2_@MGCN/GCE. The reduced resistance directly contributes to kinetics and ion diffusion, which support the higher redox currents observed in the CV studies. Therefore, the synergistic combination of MnO_2_ intercalation–deintercalation and MGCN-enhanced surface charge transfer leads to a proportional increase in anodic peak current (*I*_pa_) with Na^+^ ions.

#### 3.6.4 Effect of scan rate, pH and nanocomposite loading for Na^+^ ion detection

The effect of the scan rate on Na^+^ ion detection process in the electrochemical reaction was investigated by CV in 5 mM Na^+^ concentration in 0.1 M PBS at pH 6.5. The scan rate was varied from 25 to 250 mV s^−1^ as presented in Fig. 6(e). There was an increase in both oxidation and reduction peak currents with increasing scan rate and only a minor shift in the anodic and cathodic peak potentials was observed. In addition, the trend of anodic current (*I*_pa_) *versus* the square root of the scan rate (*ν*^1/2^) (Fig. 6(f)) was linearly expressed using a regression equation of *I*_pa_ (µA) = 0.54 *ν*^1/2^ + 0.67 and a correlation coefficient (*R*^2^) was 0.9978. Observed behaviour suggests that the electrochemical reaction is diffusion-controlled, meaning that the electrochemical reaction occurs faster toward the electrode surface with higher scan rates.

The effect of pH performance towards the electrode was also investigated in order to find the optimum pH conditions for Na^+^ ion sensing in 0.1 M PBS at a scan rate of 25 mV s^−1^. The pH range was varied from 4.5 to 8.5 with an increment of 1, as it matches the typical physiological pH of human sweat. The results obtained are displayed in Fig. 6(g), the peak current for the oxidation peak was relatively low (3.1 µA) at pH 4.5 and the peak potential for the oxidation peak was 0.22 V. Further increase in pH shows enhanced oxidation peak current and a maximum response observed at pH 6.5. The oxidation peak current was found to decrease with further increase in pH, which suggests a decrease in electrochemical response (Fig. 6(h)). In this range, the sensor showed stable and reproducible responses, with the maximum response observed at pH 6.5. Therefore, pH 6.5 was chosen as the optimum working pH for further Na^+^ sensing studies.

The effect of the Ag–MnO_2_@MGCN was further investigated by varying the drop-casting volume from 1 to 4 µL under the optimized experimental conditions (0.1 M PBS, pH 6.5 and scan rate of 25 mV s^−1^) with 10 mM of Na^+^ ions. Since the drop-casting volume directly determines the amount of nanocomposite deposited on the GCE surface and consequently affect the thickness, its optimization is essential for achieving maximum sensing performance. As shown in Fig. S6, the oxidation peak current gradually increased with increasing drop-casting volume from 1 to 3 µL, indicating enhanced electroactive surface coverage and improved electron transfer. The highest oxidation peak current was obtained at a 3 µL loading. However, a further increase to 4 µL resulted in a slight decrease in the electrochemical response, which can be attributed to the formation of a relatively thicker nanocomposite layer that hinders electrolyte diffusion and increases the electron transport pathway.^67^ Therefore, a 3 µL drop-casting volume was selected as the optimum nanocomposite loading for all electrochemical measurements.

#### 3.6.5 Quantitative detection of Na^+^ ions *via* Ag–MnO_2_@ MGCN/GCE

The Ag–MnO_2_@MGCN/GCE was used to quantitatively determine Na^+^ ions using CV and DPV. The anodic peak current increases progressively with increasing Na^+^ concentration from 5 to 35 mM in 0.1 M PBS, indicating a concentration-dependent electrochemical response (Fig. S7(a)). The high sensitivity of the electrode was verified by a linear relationship shown on the calibration plot (Fig. S7(b)) between anodic peak current and Na^+^ concentration. Additionally, a gradual shift of the anodic peak toward lower potentials was observed with increasing Na^+^ concentration, attributed due to reversible intercalation–deintercalation of Na^+^ within the MnO_2_ lattice. The detailed sensing mechanism governing this behaviour is presented in Section 3.6.3.

**Fig. 7:**
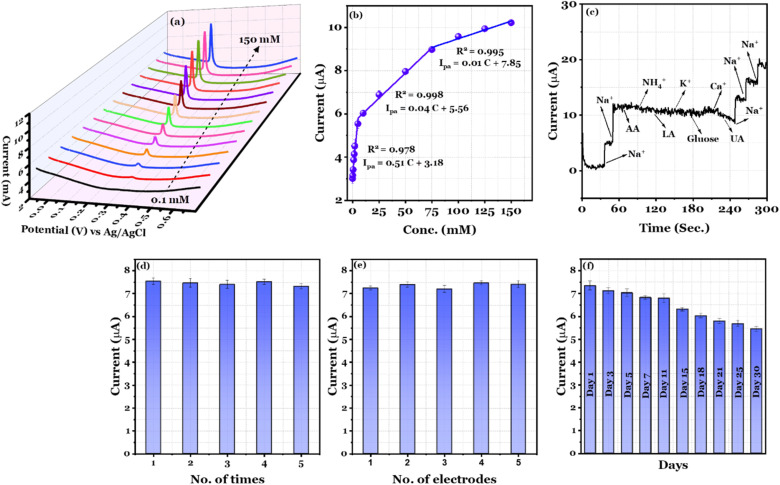
(a) DPV analysis of Ag–MnO_2_@MGCN/GCE with different Na^+^ ion concentrations ranging from 0.1–150 mM in 0.1 M PBS (pH-6.5), (b) linear plot of oxidation peak current (µA) *vs.* Concentration of Na^+^ ions (mM). (c) Amperometric response of Ag–MnO_2_@MGCN/GCE to 10 mM Na^+^ ions and different interferents in 0.1 M PBS (pH-6.5) at an applied potential of 0.3 V, (d) repeatability, (e) reproducibility, and (f) stability of Ag–MnO_2_@MGCN/GCE towards 25 mM in 0.1 PBS (pH-6.5).

Further DPV studies were conducted under optimized conditions to quantitatively evaluate Na^+^ sensing performance. The oxidation peak current shows a significant linear increase from 0.1 to 150 mM of Na^+^ concentration, as indicated in Fig. 7(a). The corresponding calibration plot (Fig. 7(b)) reveals three distinct linear response regions of 0.1 to 5 mM, 5 to 75 mM, and 75 to 150 mM. The multi-linear calibration is attributed to the concentration-dependent kinetics of the reversible Na^+^ intercalation–deintercalation process coupled with the Mn^3+^/Mn^4+^ redox transition. At low Na^+^ concentrations, abundant electrochemically accessible MnO_2_ sites facilitate rapid Na^+^ intercalation–deintercalation, resulting in a higher current response and a steeper calibration slope. As the Na^+^ concentration increases, progressive occupation of the electrochemically accessible MnO_2_ sites reduces the availability of active sites, leading to a decrease in sensitivity and the second linear response becomes increasingly influenced by Na^+^ diffusion limitations together with partial saturation of the calibration slope. Further, a third linear region arises from the combined influence of concentration-dependent Na^+^ intercalation–deintercalation kinetics, diffusion limitations, and partial saturation of the electroactive MnO_2_ sites. The corresponding linear regression equations and correlation coefficient (*R*^2^) for all three concentration ranges demonstrated good linearity (Fig. 7(b)). The analytical parameters, such as limits of detection (LOD), limits of quantification (LOQ), and sensitivity, for all three concentration ranges are summarized in Table S2. At the same time, the detailed calculation procedures for LOD, LOQ and sensitivity are provided in the SI (Section S1). The LOD values for the three concentration ranges were 0.06 mM, 0.86 mM, and 3.42 mM, respectively, confirming the high analytical performance of the Ag–MnO_2_@MGCN/GCE sensor.

A comparative study (Table 1) with previously reported Na^+^ ion sensors highlights the improved analytical performance of the present work. The sensor exhibited a broader linear detection range (0.1–150 mM) compared to Ag NPs/GO/SPE (10–100 mM), Cu–ZnO/GCE (8.7 × 10^−4^–4.35 × 10^−2^ mM), and Na_0.44_MnO_2_ (0.21–24.54 mM), enabling Na^+^ detection over a wide concentration window.^68–70^ Further, the lowest limit of detection achieved was 0.06 mM in the 0.1 to 5 mM range and the sensitivity (9.62 µA mM^−1^ cm^−2^) was higher than that for Ag NPs/GO/SPE (0.269 µA mM^−1^ cm^−2^) and Cu–ZnO/GCE (1.023 µA mM^−1^ cm^−2^).^68,69^ However, FePO_4_/GCE has a wide detection range (25–200 mM) and Ag–MnO_2_@MGCN/GCE offers a good detection range and sensitivity.^17^ The utilization of DPV is further improved detection resolution than CV and LSV based sensors, which validates the applicability of Ag–MnO_2_@MGCN/GCE sensor for sensitive Na^+^ ion sensing.

**Table 1:** Comparison of our proposed electrode with the previously published articles^a^

Sr. no.	Modified electrode	Method	Linear range (mM)	LOD (mM)	Sensitivity (mA mM^−1^ cm^−2^)	Ref.
01	Ag NPs/GO/SPE	CV	10–100	9.344	0.269	71
02	Cu–ZnO/GCE	CV	8.7 × 10^−4^–4.35 × 10^−2^	8.7 × 10^−4^	0.001	72
03	Hausmannite-type MnO_2_/GCE	CV	0.02–0.2	0.0075	—	65
04	FePO_4_/GCE	CV	25–200	—	—	17
		LSV				
05	Na_0.44_MnO_2_	PM	0.21–24.54	—	—	70
06	Ag–MnO_2_@MGCN/GCE	DPV	0.1–5, 5–75, & 75–150	0.06, 0.85, & 3.42	9.62, 0.75, & 0.18	Present work

a CV-cyclic voltammetry, DPV-differential pulse voltammetry, LSV-linear sweep voltammetry, and PM-potentiometry.

#### 3.6.6 Selectivity, repeatability, reproducibility, and stability studies

The selectivity of the Ag–MnO_2_@MGCN/GCE sensor was verified using chronoamperometric (CA) measurements in the presence of common interferents including ascorbic acid (AA), glucose, lactic acid (LA), uric acid (UA), potassium (K^+^), ammonium (NH_4_^+^), and calcium (Ca^2+^). In the experiment, 10 mM Na^+^ ions were added sequentially, followed by 10-fold excess of each interferent and then Na^+^ ions were added into 0.1 M PBS (pH-6.5) with constant potential of 0.3 V. The sensor exhibited stable response toward Na^+^ ions, while the presence of interfering species induced negligible current change, indicating the sensor's high selectivity.

In addition, DPV measurements were performed to further evaluate the selectivity of the developed sensor. The DPV response was recorded for Na^+^(25 mM), followed by the sequential addition of the above interfering species, together with Mg^2+^ and Cl^−^, each at a concentration 10-fold higher than Na^+^. Additionally, 25 mM Na^+^ was reintroduced to evaluate the electrochemical response (Fig. S8(a)). The presence of the interfering analytes showed negligible changes in the oxidation peak potential and peak current, whereas the re-addition of Na^+^ exhibited an increase in the oxidation peak current, confirming the excellent selectivity of the Ag–MnO_2_@MGCN/GCE sensor toward Na^+^ ions. Furthermore, the corresponding recovery values (Table S3) demonstrated analytical reliability in the presence of coexisting ionic and molecular species. These results confirm the high selectivity of Ag–MnO_2_@MGCN/GCE sensor for Na^+^ ion detection in complex biological environments like sweat.

The repeatability, reproducibility, and stability of Ag–MnO_2_@MGCN/GCE sensor was systematically evaluated using DPV using 0.1 M PBS (pH 6.5) with 25 mM Na^+^ ions. Repeatability was tested by taking five successive DPV scans with the same electrode under the same experimental conditions. The sensor showed a stable reduction peak current during all cycles, along with RSD (relative standard deviation) of 1.21% (Fig. 7(d) and S9(a)). The consistent current response suggests the electrode surface remains stable, with no significant degradation or fouling across repeated use.

Five electrodes were independently fabricated and tested to validate reproducibility of Ag–MnO_2_@MGCN/GCE sensor, where the RSD was 1.5%. This low RSD confirms that minimal variation across electrodes, which means that the sensor is high reproducible (Fig. 7(e) and S9(b)). The long-term stability investigated by measuring the oxidation peak current for 30 days, and by storing the Ag–MnO_2_@MGCN/GCE electrode at room temperature in a desiccator. The sensor retained 74.25% of its initial current response after 30 days, demonstrating satisfactory storage stability and sustained electrochemical performance (Fig. 7(f) and S9(c)). The results collectively demonstrate that the Ag–MnO_2_@MGCN/GCE sensor has good repeatability, reproducibility, and stability, making it a reliable platform for Na^+^ ion detection.

#### 3.6.7 Recovery studies of Na^+^ ions in artificial sweat samples

The artificial sweat sample was used to investigate the practical applicability of the Ag–MnO_2_@MGCN/GCE sensor, and artificial sweat sample was prepared using the protocol reported in our previous work.^22,73^ Before electrochemical analysis, the sweat sample diluted 10-fold with 0.1 M PBS (pH 6.5) to obtain a stable and reproducible electrochemical response. CV measurements recorded for the diluted artificial sweat sample (Fig. S10(a)) exhibit an oxidation peak that increases systematically with increasing Na^+^ concentration, ranging from 10 to 30 mM. Recovery values of Na^+^ ions are summarized in Table 2. The obtained recovery values ranged from 96.46% to 101.2%, demonstrating the good accuracy and repeatability. Furthermore, the Na^+^ concentrations determined using the Ag–MnO_2_@MGCN/GCE sensor showed good agreement with those obtained by ICP-OES, further validating the analytical reliability of the proposed sensing for Na^+^ quantification in artificial sweat.

**Table 2:** Recovery analysis of Na^+^ ions in artificial sweat using the Ag–MnO_2_@MGCN/GCE (*n* = 3)

Sample	Added (mM)	Electrochemical sensor		ICP-OES	
		Detected (mM)	Recovery (%)	Detected (mM)	Recovery (%)
Artificial sweat	10	10.12	101.2	10.21	102.1
	20	19.48	97.4	19.64	98.2
	30	28.94	96.46	29.36	97.9

## 4 Conclusion

A highly sensitive and selective electrochemical sensor for the detection of Na^+^ ions was developed using Ag–MnO_2_@MGCN as the sensing material. The sensor showed excellent electrochemical performance with a distinct redox response with oxidation and reduction peaks at 0.25 V and 0.08 V, respectively. The sensor exhibited high sensitivity and good reproducibility with a wide linear detection range of 0.1 to 150 mM and a LOD of 0.06 mM. The unique sensing mechanism is based on the intercalation–deintercalation of Na^+^ ions within the MnO_2_ matrix and surface charge transfer facilitated by Ag NPs and MGCN, which enhances electron transfer kinetics. The sensor's potential application was evaluated through the detection of Na^+^ ions in an artificial sweat sample with high recovery efficiency and reliability. The Ag–MnO_2_@MGCN/GCE sensor demonstrated a promising platform for noninvasive Na^+^ ion monitoring, with potential applications in wearable health-monitoring devices. Future research may focus on integrating this sensor into flexible substrates for real-time, portable, and continuous monitoring in healthcare applications.

## Author contributions

Nikita J. Patil: conceptualization, methodology, formal analysis, investigation, data collection, writing – original draft, writing – review & editing. Ganesh Kumar Mani: methodology, writing – review & editing. K. Kailasam: conceptualization, supervision, writing review, and editing. Murali Rangarajan: supervision, conceptualization, methodology, writing – review & editing. Parthasarathy Srinivasan: supervision, funding acquisition, conceptualization, methodology, writing – review & editing.

## Conflicts of interest

The authors declare no competing financial interest.

## Supplementary Material

RA-OLF-D6RA04109D-s001

## Data Availability

The additional data supporting this article have been presented as part of the supplementary information (SI). Supplementary information is available. See DOI: https://doi.org/10.1039/d6ra04109d.
