# Supplementary material for: Investigation of a surface decorated Ag–MnO2@MGCN nanointerface towards membrane-free electrochemical Na+ ion sensing in sweat
Source: RSC Adv. 2026 Aug 18;16(41):48499–512. doi: 10.1039/d6ra04109d (PMC13482744; doi:10.1039/d6ra04109d)
Supplement: Supplementary file 1 [file RA-016-D6RA04109D-s001.pdf]

Supplementary Information (SI) for RSC Advances.
This journal is © The Royal Society of Chemistry 2026

# Investigation on Surface Decorated Ag-$MnO_2$@MGCN Nanointerface Towards Membrane-Free Electrochemical Sweat $Na^+$ Ion Sensing

***Nikita J. Patil [a,b], Ganesh Kumar Mani[c], Kamalakannan Kailasam[d*], Murali Rangarajan[b,e,f*], Parthasarathy Srinivasan[a*]***

[a] *Center of Excellence in NanoTechnology & Advanced Materials (CENTAM), Department of Electronics and Communication Engineering, Amrita School of Engineering, Amrita Vishwa Vidyapeetham, Chennai, 601103, Tamil Nadu, India*

[b] *Center of Excellence in Advanced Materials and Green Technologies, Amrita School of Engineering Coimbatore, Amrita Vishwa Vidyapeetham, India.*

[c] *Department of Physics, Korea Advanced Institute of Science and Technology, Korea.*

[d] *Advanced Functional Nanomaterials, Institute of Nano Science and Technology (INST), Knowledge City, Sector-81, Manauli, SAS Nagar, 140306 Mohali, Punjab, India.*

[e] *Department of Chemical Engineering and Materials Science, Amrita School of Engineering Coimbatore, Amrita Vishwa Vidyapeetham, India.*

[f] *Gurukripa Electrolyzers Private Limited, Coimbatore, India.*

*** Corresponding Authors:** r_murali@cb.amrita.edu and s_parthasarathy@ch.amrita.edu

**S.1 Calculation of standard deviation of blank ($SD_b$):**

**Standard deviation (SD) formula:** $$s = \sqrt{\frac{\sum_{i=1}^{n} (x_i - \bar{x})^2}{n - 1}}$$

| Blank sample | Current value of blank solution (μA) |
|---|---|
| $B_1$ | 2.72 |
| $B_2$ | 2.71 |
| $B_3$ | 2.70 |
| $B_4$ | 2.69 |
| $B_5$ | 2.70 |
| Statistics | Mean, $\bar{B}$ = 2.704 |
| | $SD_b$ = 0.0114 |

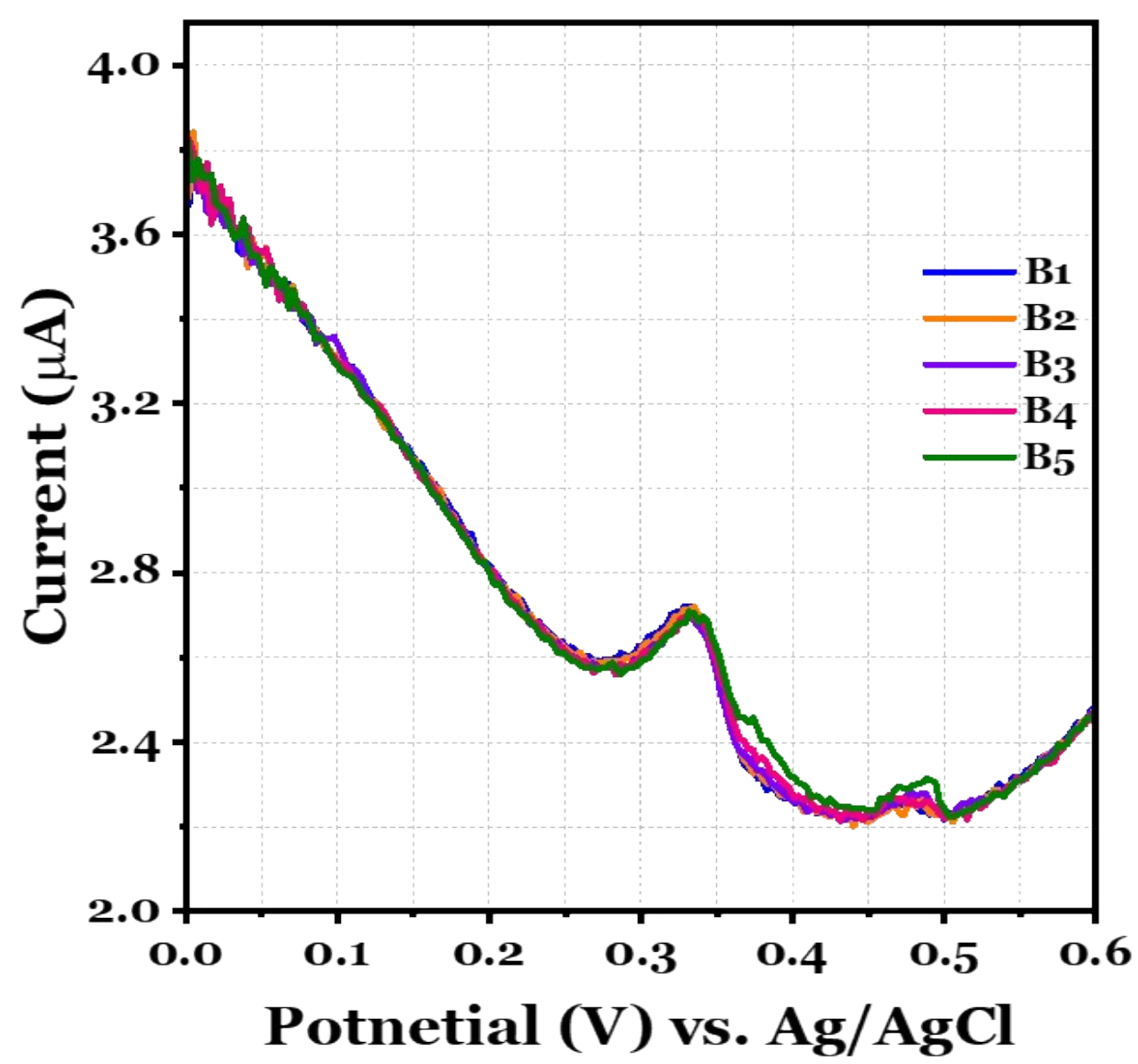


$$LOD = 3\frac{SD}{m}$$

- For 0.5 to 5 mM range

  LOD = (3*0.0114)/0.51

  LOD = 0.06 mM

- For 5 to 75 mM range

  LOD = (3*0.0114)/0.04

  LOD = 0.85 mM

- For 75 to 150 mM range

  LOD = (3*0.0114)/0.01

  LOD = 3.42 mM

$$LOQ = 10\frac{SD}{m}$$

- For 0.5 to 5 mM range

  LOD = (10*0.0114)/0.51

  LOD = 0.22 mM

- For 5 to 75 mM range

  LOD = (10*0.0114)/0.04

  LOD = 2.85 mM

- For 75 to 150 mM range

  LOD = (3*0.0114)/0.01

  LOD = 11.4 mM

Here, SD = standard deviation of the blank and m = slope.

$$Sensitivity = \frac{B}{A}$$

- For 0.5 to 5 mM range

  Sensitivity = 0.51 / 0.053

  Sensitivity = 9.62 $\mu A/mM.cm^{2-}$

- For 5 to 75 mM range

  Sensitivity = 0.04 / 0.053

  Sensitivity = 75 × $10^{-2}$ $\mu A/mM.cm^{2-}$

- For 75 to 150 mM range

  Sensitivity = 0.01 / 0.053

  Sensitivity = 188 × $10^{-3}$ $\mu A/mM.cm^{2-}$

Here, b = slope of the calibration plot and A = electroactive surface area of the electrode.

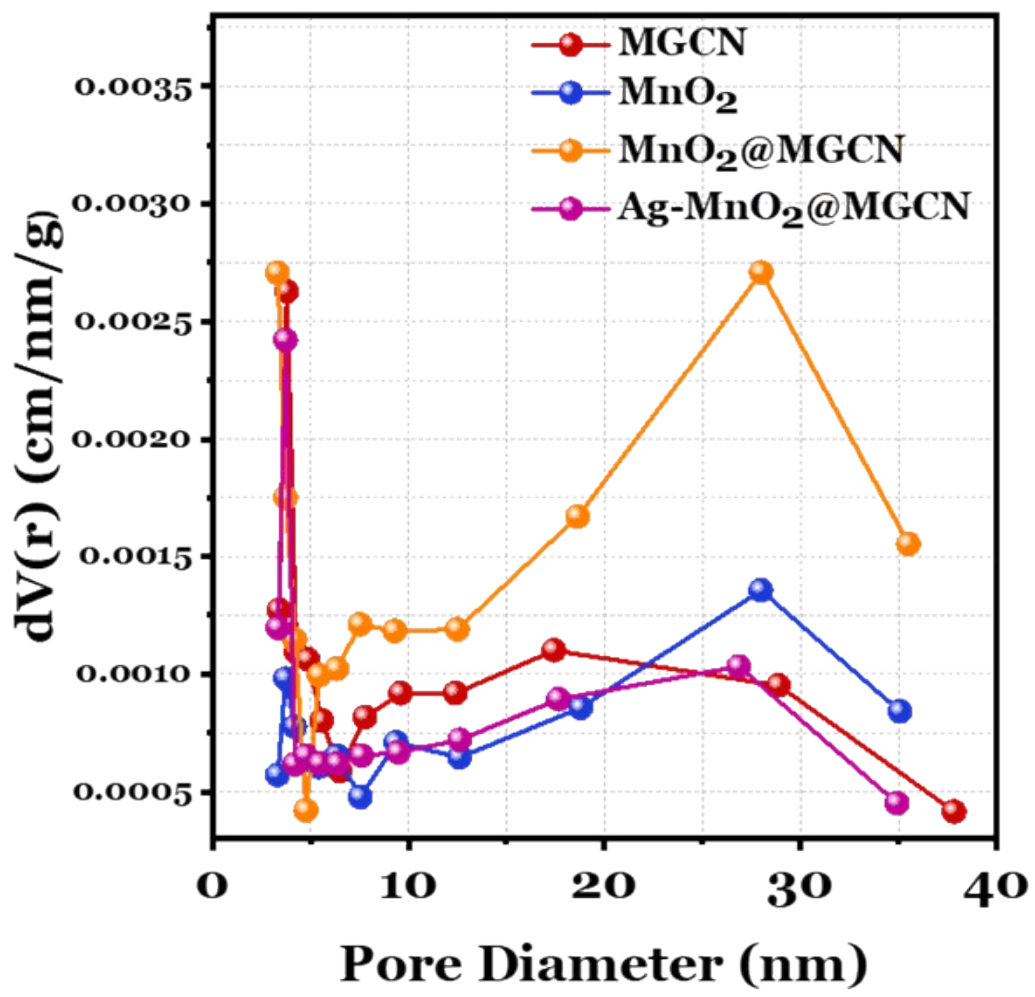


Fig. S1 Pore size distribution of MGCN, $MnO_2$, $MnO_2$@MGCN, and Ag-$MnO_2$@MGCN

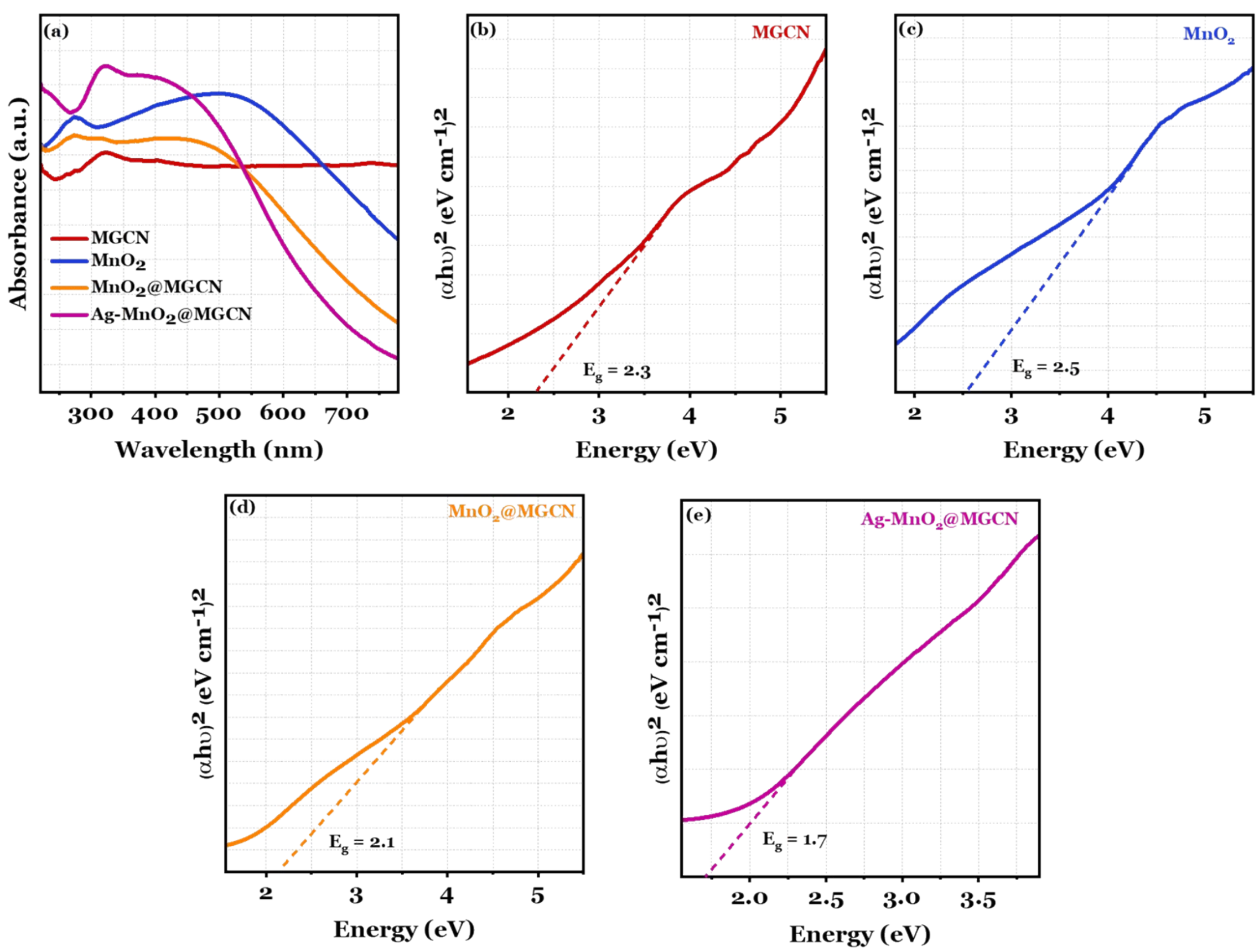


Fig. S2 (a) UV-Vis absorption spectra, and (b-e) Tauc plot of MGCN, $MnO_2$, $MnO_2$@MGCN, and Ag-$MnO_2$@MGCN.

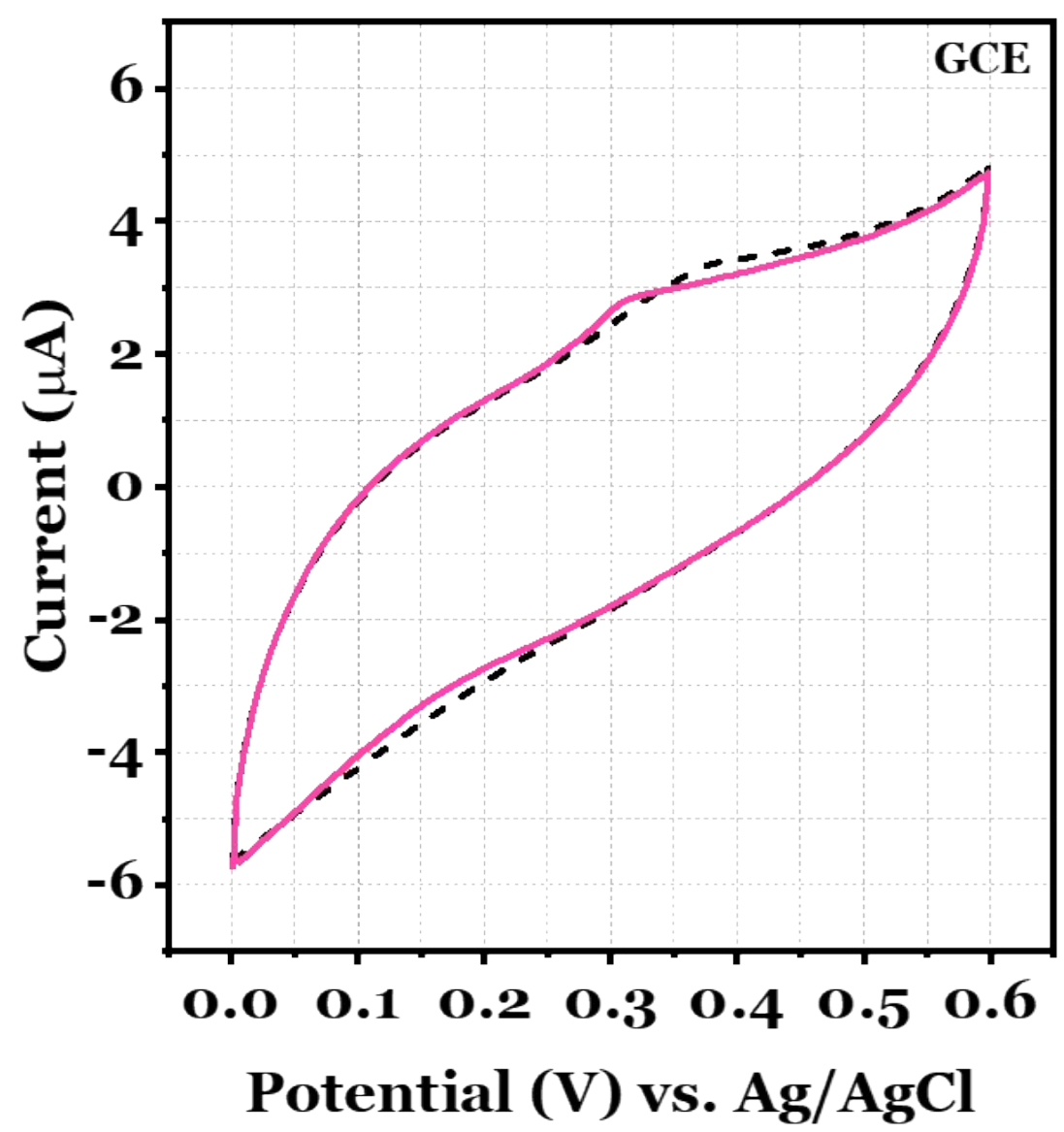


Fig. S3 CV curve of GCE in 0.1 M PBS (pH-6.5), absence (dotted line) and presence (solid line) of 5 mM $Na^{+}$ ions at the scan rate of 25 mV $s^{-1}$.

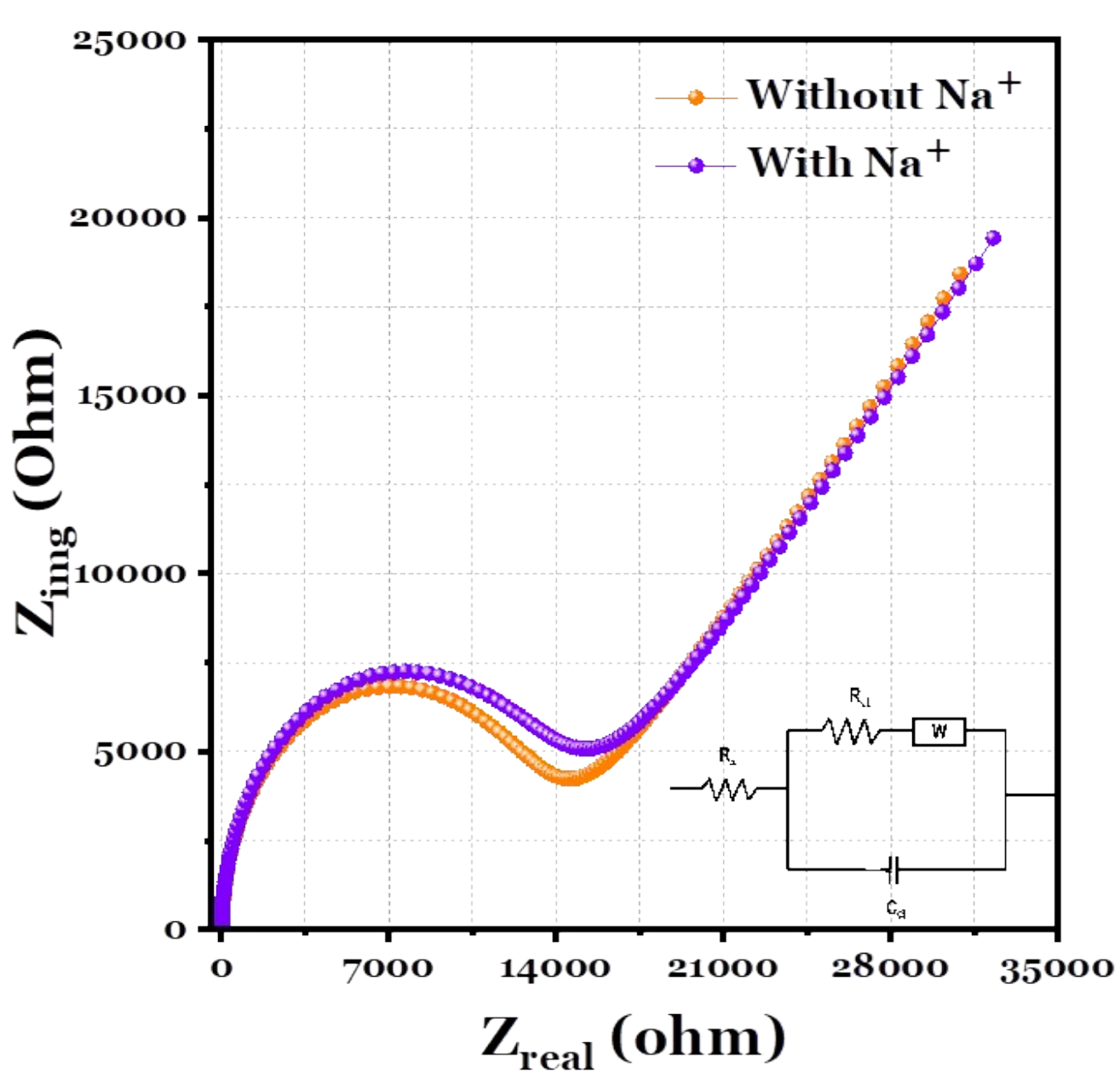


Fig. S4 Nyquist impedance spectra of Ag-$MnO_2$@MGCN/GCE before and after $Na^+$ exposure with the corresponding equivalent circuit (inset).

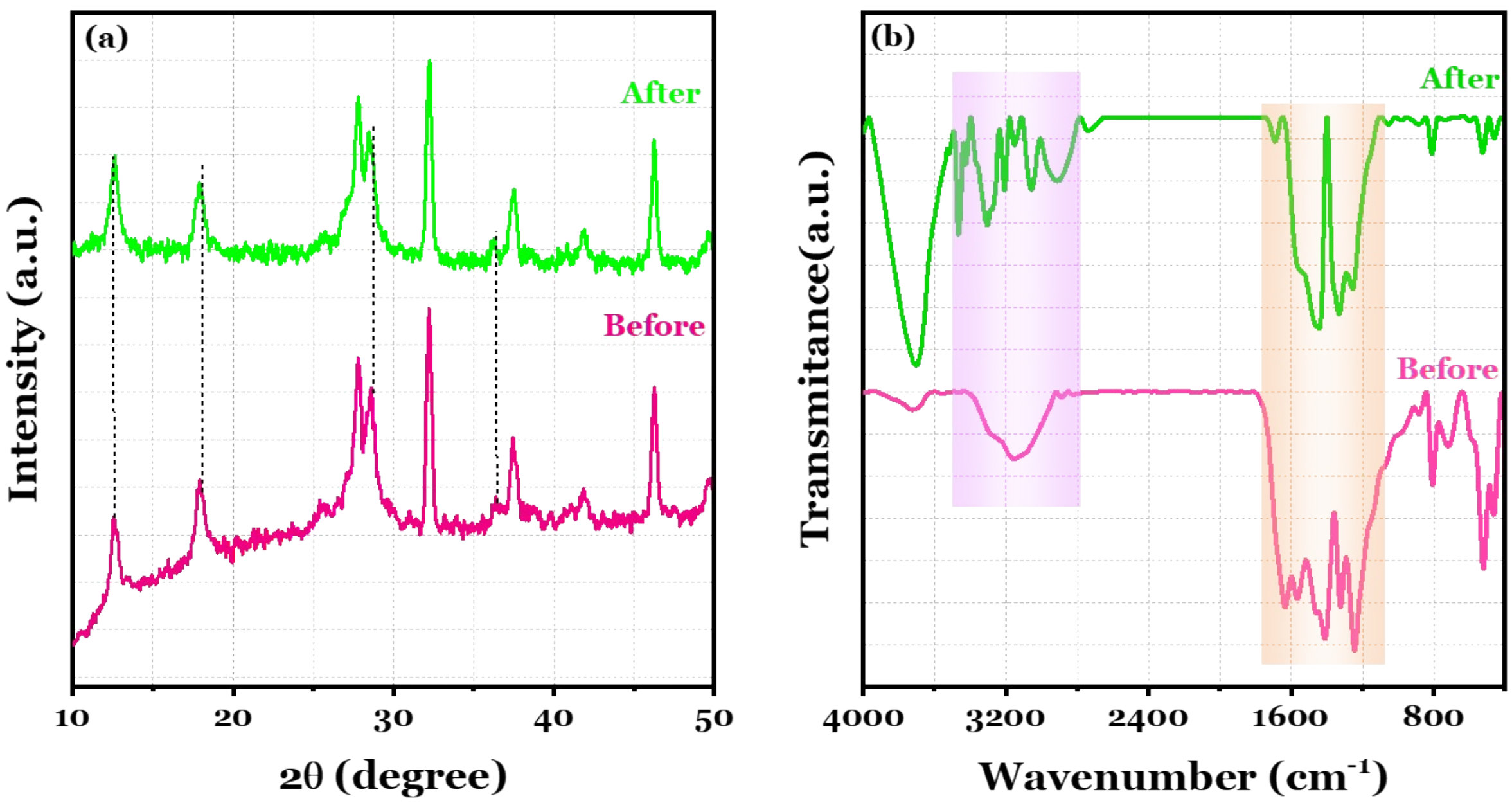


Fig. S5 (a) XRD patterns and (b) FTIR spectra of the Ag-$MnO_2$@MGCN nanointerface before and after $Na^+$ treatment.

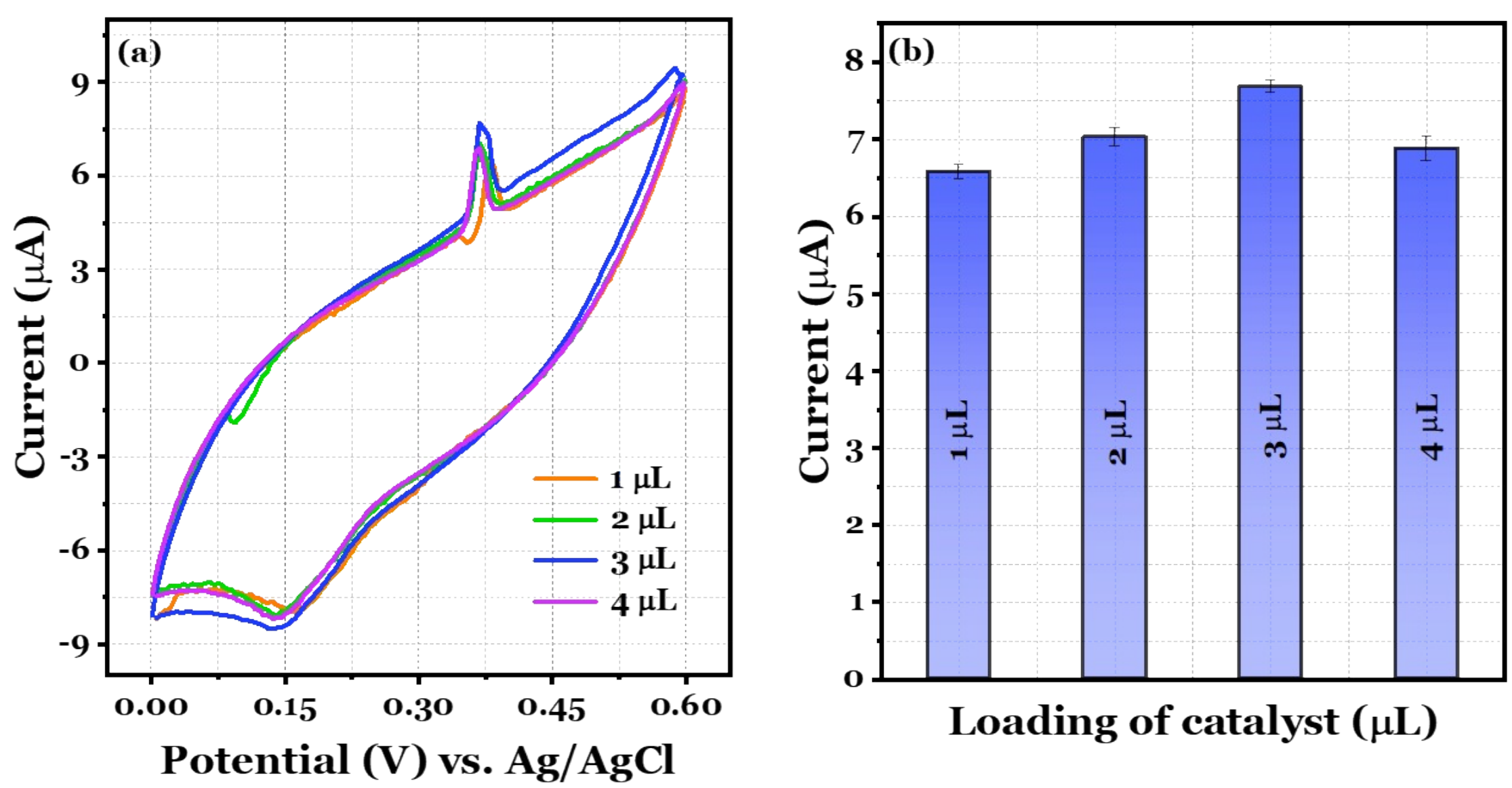


Fig. S6 (a) CV curves of Ag-$MnO_2$@MGCN/GCE recorded with different catalyst loading volumes and (b) the corresponding oxidation peak current as a function of catalyst loading.

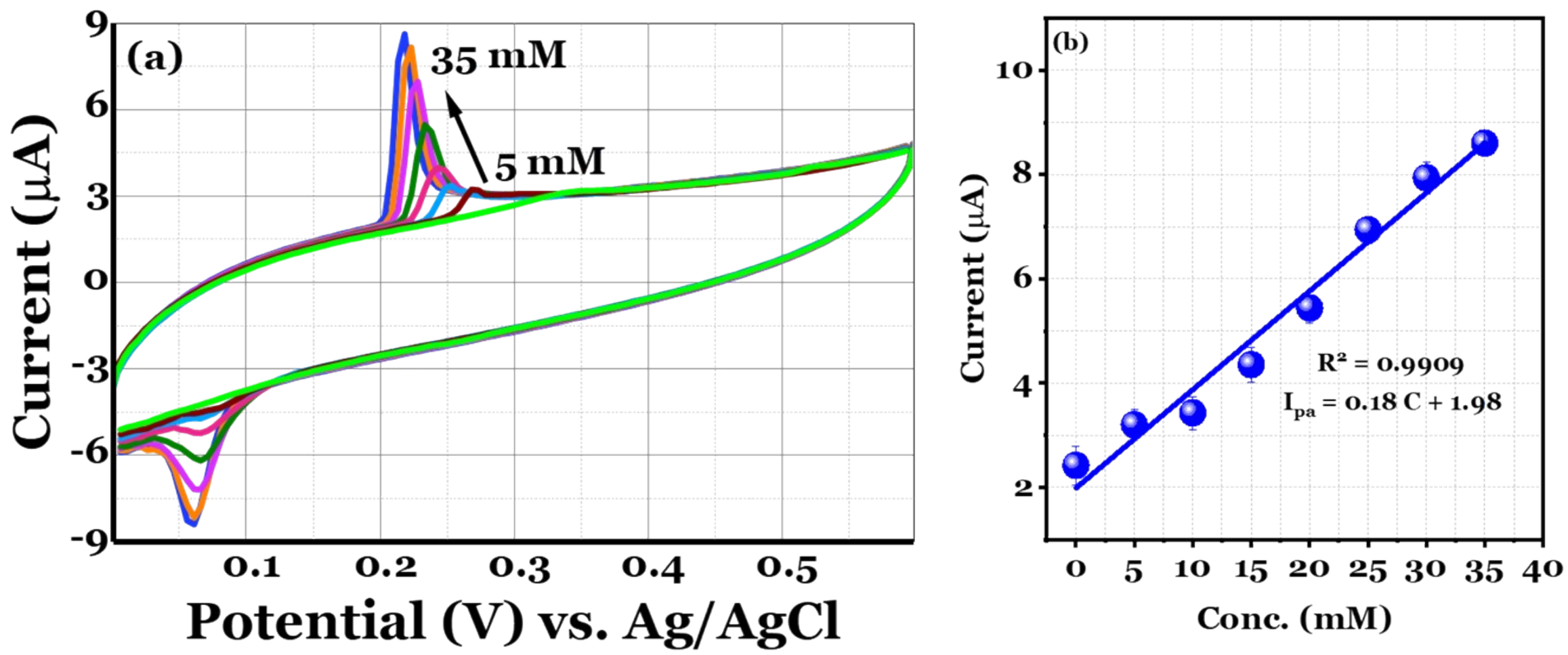


Fig. S7 (a) CV response of Ag-$MnO_2$@MGCN/GCE in the presence of different concentration of $Na^+$ ions from 5 to 35 mM in 0.1 M PBS (pH-6.5) at scan rate of 25 mV $s^{-1}$, (b) linear plot of oxidation current (μA) versus $Na^+$ ions concentration (mM)

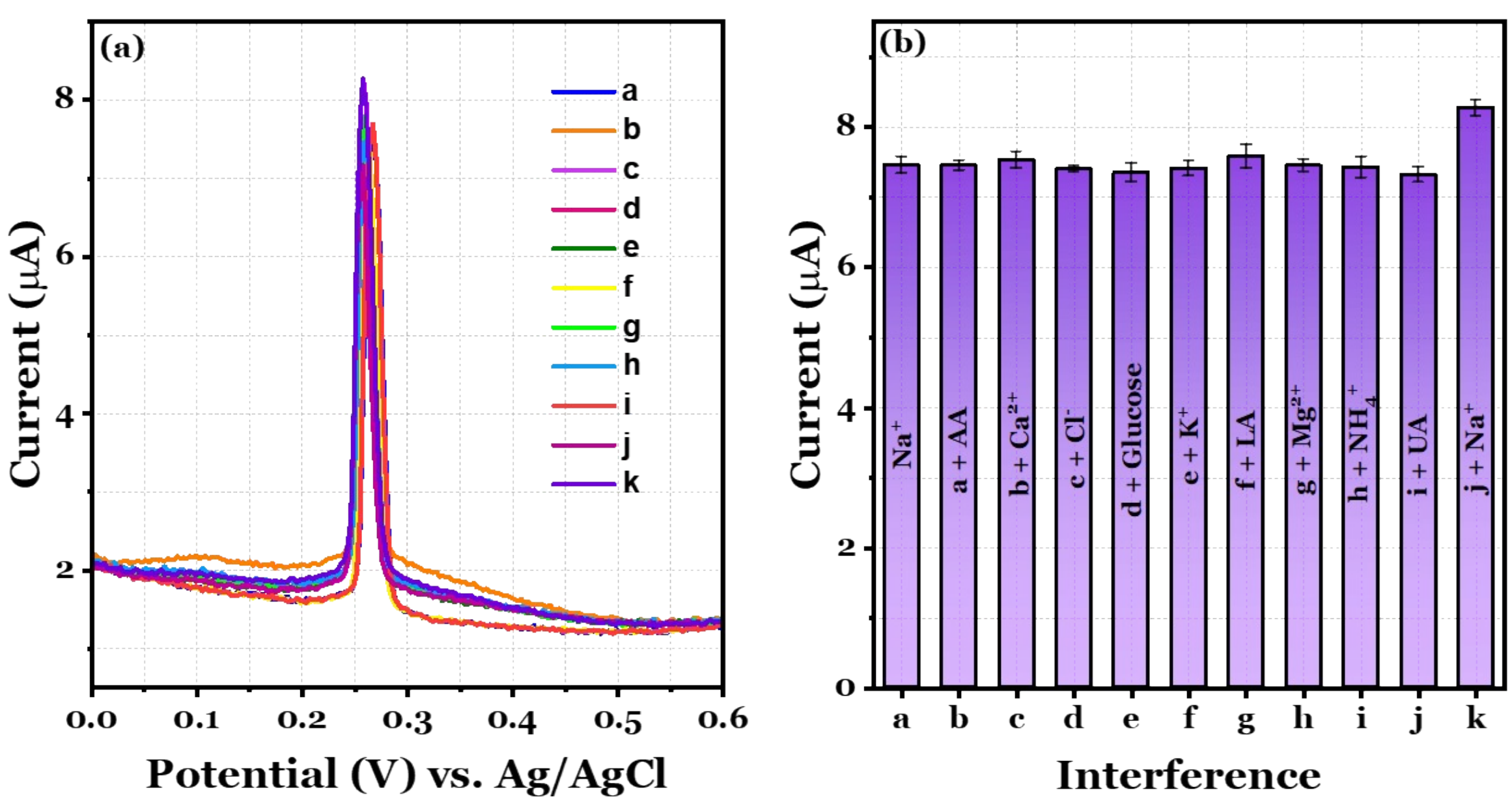


Fig. S8 (a) DPV selectivity response of Ag-$MnO_2$@MGCN/GCE toward 25 mM $Na^+$ ions in the presence of common interfering analytes in 0.1 M PBS (pH 6.5) followed by re-addition of $Na^+$. (b) Corresponding oxidation peak current responses.

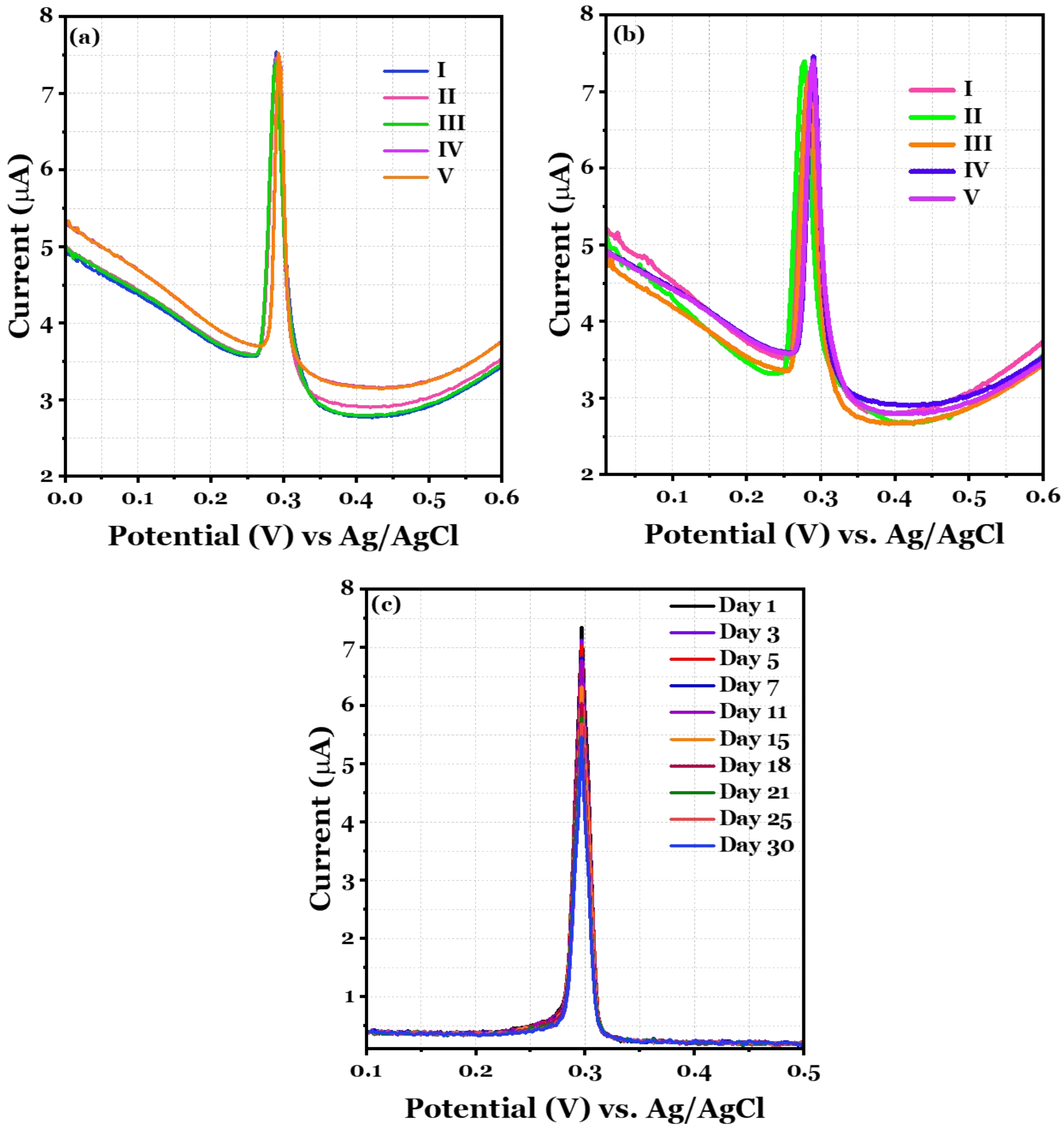


Fig. S9. DPV response of Ag-$MnO_2$@MGCN/GCE in the presence of 25 mM of $Na^+$ ions in 0.1 M PBS (pH-6.5) for (a) repeatability analysis, (b) reproducibility analysis, and (c) stability analysis.

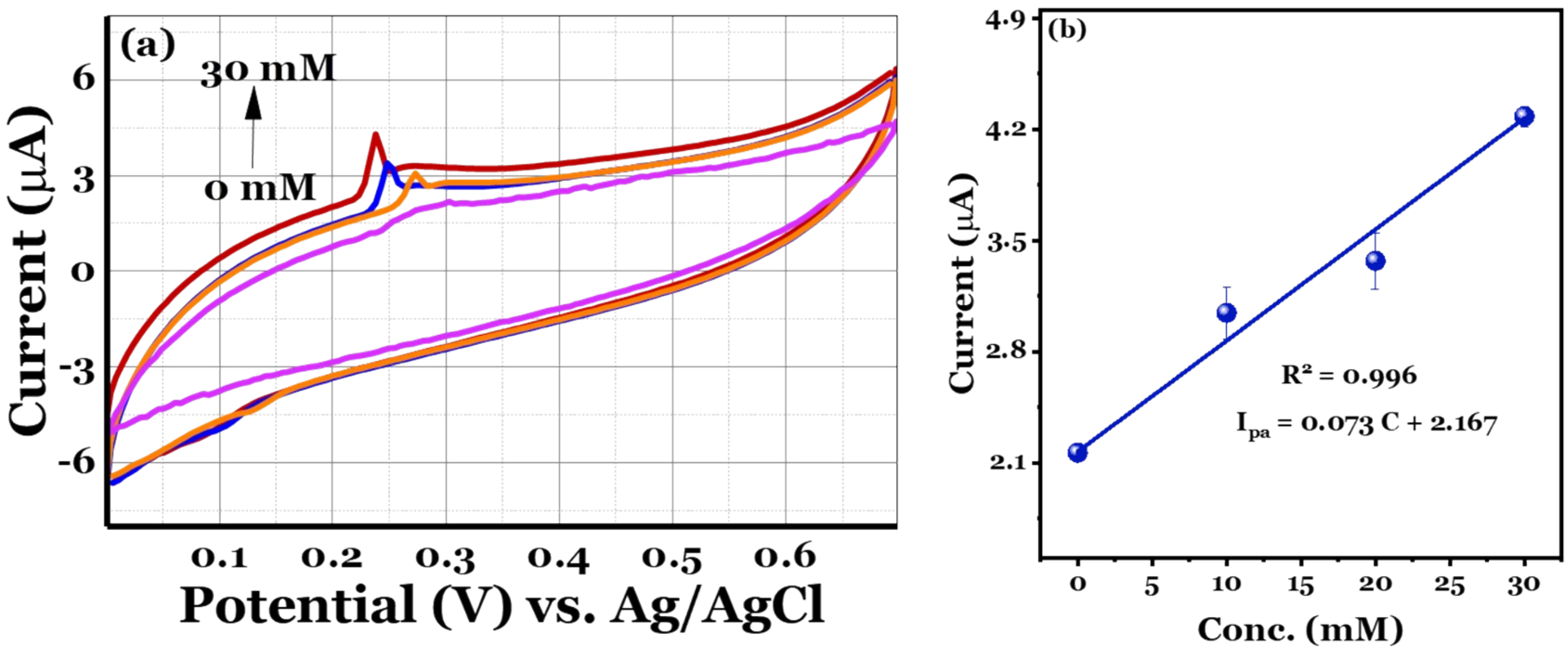


Fig. S10 (a) CV profile of the Ag-$MnO_2$@MGCN/GCE in an artificial sweat sample, and (b) the corresponding calibration plot for the artificial sweat sample.

| Electrode | $R_{ct}$ (kΩ) |
|---|---|
| Bare GCE | 1.90 |
| MGCN/GCE | 0.94 |
| $MnO_2$/GCE | 0.42 |
| $MnO_2$@MGCN/GCE | 0.46 |
| Ag-$MnO_2$@MGCN/GCE | 0.40 |

**Table S1.** $R_{ct}$ values of bare and modified electrodes derived from EIS spectra

**Table S2**. Analytical parameters of the Ag-$MnO_2$@MGCN/GCE sensor for $Na^+$ detection.

| Linear range (mM) | Slope ($\mu A\ mM^{-1}$) | LOD (mM) | LOQ (mM) | Sensitivity ($\mu A\ mM^{-1}\ cm^{-2}$) |
|---|---|---|---|---|
| 0.5 – 5 | 0.51 | 0.06 | 0.22 | 9.62 |
| 5 – 75 | 0.04 | 2.85 | 2.85 | 0.75 |
| 75 – 150 | 0.01 | 3.42 | 11.4 | 0.188 |

**Fig. S3** Recovery performance of the Ag-$MnO_2$@MGCN/GCE sensor for $Na^+$ ion determination in the presence of typical interfering species

| Sr. No. | Sample | Analyte | Peak Current (μA) | Recovery (%) |
|---|---|---|---|---|
| 01 | a | $Na^+$ | 7.46 | - |
| 02 | b | a + AA | 7.45 | 99.86 |
| 03 | c | b + $Ca^{2+}$ | 7.53 | 100.93 |

| | | | | |
|---|---|---|---|---|
| 04 | d | c + $Cl^-$ | 7.40 | 99.19 |
| 05 | e | d + Glucose | 7.35 | 98.52 |
| 06 | f | e + $K^+$ | 7.41 | 99.32 |
| 07 | g | f + LA | 7.58 | 101.60 |
| 08 | h | g + $Mg^{2+}$ | 7.45 | 99.86 |
| 09 | i | h + $NH_4^+$ | 7.43 | 99.59 |
| 10 | j | i + UA | 7.32 | 98.12 |
| 11 | k | j + $Na^+$ | 8.27 | - |
